# Synthesis of 2,6-*trans*-disubstituted 5,6-dihydropyrans from (*Z*)-1,5-*syn*-endiols

**DOI:** 10.1186/1860-5397-1-7

**Published:** 2005-08-26

**Authors:** Eric M Flamme, William R Roush

**Affiliations:** 1 Department of Chemistry, University of Michigan, Ann Arbor, MI 48109, USA; 2 Current Address: Department of Chemistry, Princeton University, Princeton, NJ 08544, USA; 3 Current Address: Department of Medicinal Chemistry, Scripps Florida, Jupiter, Fl 33458, USA

## Abstract

Certain (*Z*)-1,5-*syn*-diols **2** may be converted into 2,6-*trans*-5,6-dihydropyrans by using phosphonium salt **4** or phosphorane **5** as dehydrating agents. A more general four step procedure converts the (*Z*)-1,5-*syn*-endiols into enantiomeric dihydropyrans *ent*-**3** via regioselective silylation of the allylic alcohol unit followed by mesylate formation and base-promoted nucleophilic displacement.

## Findings

**Scheme 1:**
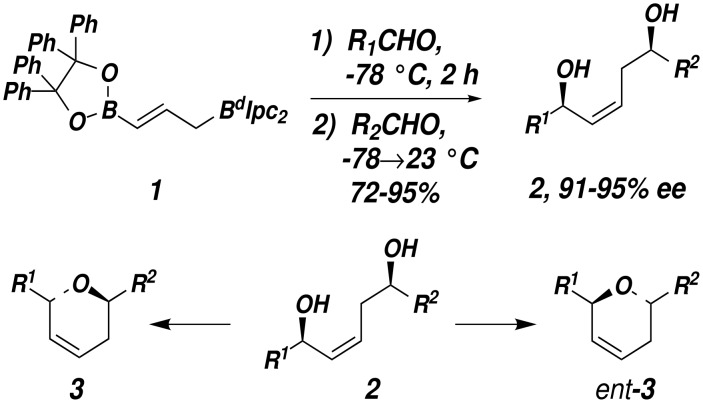


We recently reported a one-pot double allylboration reaction sequence which provides (*Z*)-1,5-*syn*-endiols from simple aldehyde starting materials with excellent diastereo- and enantioselectivity.[1] In connection with an ongoing natural product synthesis project, we were interested in developing methods to transform diols **2** into dihydropyrans **3** or the enantiomeric dihydropyrans *ent*-**3** through complementary, regioselective cyclodehydration processes (Scheme 1).

2,6-Disubstituted dihydropyrans are common structural elements of many biologically active natural products.[2–3] A number of methods have been developed to synthesize substituted dihydropyrans including: (i) hetero-Diels-Alder cycloadditions,[4–7] (ii) electrophile-initiated alkylation of glycals,[8–11] (iii) ring closing metathesis,[12–13] (iv) vinylsilane cyclization of oxocarbenium ions,[14] and (v) intramolecular allylations.[15–16] However, we were unaware of any reports that describe the direct conversion of 1,5-diols containing an internal olefin such as **2** directly to 2,6-*trans*-disubstituted 5,6-dihydropyrans. Furthermore, there are only limited reports describing the stereoselective synthesis of related tetrahydropyrans through cyclodehydration of enantiopure 1,5-diols substrates.[17]

The challenge of synthesizing dihydropyrans **3** or *ent*-**3** from 1,5-diols such as **2** lies in the differentiation of the two hydroxyl groups. Selective activation of the allylic alcohol in **2** as a leaving group followed by nucleophilic attack by the homoallylic alcohol will lead to dihydropyran **3**. However, activation of the homoallylic alcohol followed by nucleophilic attack by the allylic alcohol will provide the enantiomeric dihydropyan *ent*-**3**. Cyclic ethers of various ring size have been synthesized by the cyclodehydration of diols through the use of oxyphosphonium salts and phosphorane reagents.[18–23] We reasoned that because the rate determining step of these cyclizations is believed to be the nucleophilic substitution step, selective formation of **3** should be possible owing to the superior leaving group ability of the activated allylic hydroxyl.[21]

**Figure 1:**
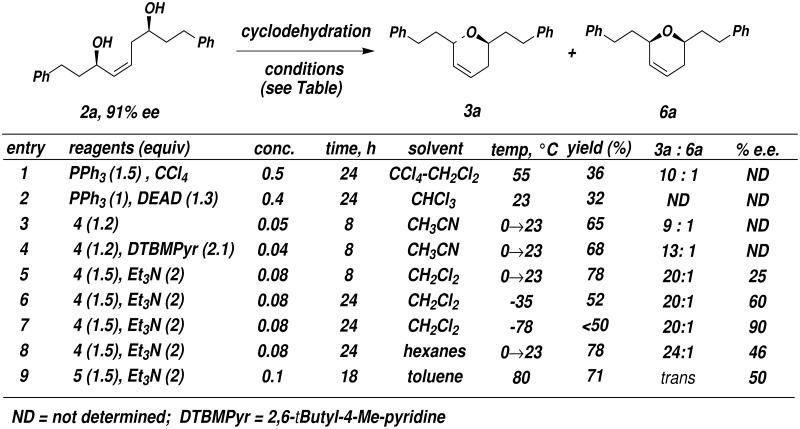
Cyclodehydration of diol **2a**

**Scheme 2:**
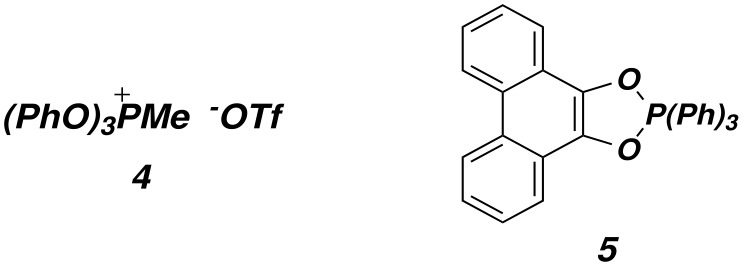


Diol **2a** (R_1_ = R_2_ = CH_2_CH_2_Ph) was used initially in the development of a suitable cyclodehydration protocol (Figure 1). Because the R_1_ and R_2_ substituents of **2a** are identical, steric effects on the activation of the two hydroxyl groups are eliminated. Therefore, the enantioselectivity of the ring closing step will depend only on the relative rates of the competing cyclization processes leading to **3a** and *ent*-**3a**. Initial attempts at cyclization of **2a** using Ph_3_P and diethyl azodicarboxylate or Ph_3_P-CCl_4_ were low yielding (entries 1, 2).[22] Interestingly, small amounts of the 2,6-*cis*-disubstituted dihydropyran **6a** were detected under these conditions, suggesting the intervention of a competitive double inversion process or a carbocation-mediated cyclization process. In order to avoid the presence of nucleophilic counter ions, we turned to the use of phosphonium salt **4** and phosphorane **5** as the cyclodehydration reagents (Scheme 2).[24–25]

Treatment of diol **2a** with **4** in acetonitrile (0.05 M) at 0°C and warming slowly to 23°C provided a 9 : 1 mixture of *trans* and *cis* dihydropyrans **3a** and **6a** in 65% combined yield (entry 3). We speculated that the *cis* isomer might arise via formation of an allylic cation (perhaps facilitated by acid formed by competitive elimination of the oxyphosphonium salt during the reaction). Accordingly, the *trans*/*cis* ratio was improved to 13 : 1 by addition of 2,6-*tert*-butyl-4-methyl-pyridine as an acid scavenger (entry 4). Ultimately, we found that use of CH_2_Cl_2_ as the solvent and Et_3_N as base provided **3a** in 78% yield with 20:1 *trans*/*cis* selectivity (entry 5). However, chiral HPLC analysis indicated that **3a** from entry 5 had an enantiomeric excess of only 25% e.e. while the enantiomeric purity of the starting diol **2a** was 91% e.e.

This result suggests that the selectivity for displacement of the two hydroxyl groups of **2a** is only ca. 2 : 1 under these conditions. Examination of molecular models indicates that in order for the activated allylic alcohol to be displaced in this intramolecular substitution process, the allylic C-O bond substantially deviates from coplanarity with the adjacent π-system. Therefore, the difference in relative rates of displacement of the two activated hydroxyl groups is much less than originally anticipated. The enantioselectivity could be increased to 90% by lowering the reaction temperature to -78°C, but the yield of dihydropyran **3a** was reduced to less than 50% (entry 7).

Dehydration of **2a** with phosphorane **5** in toluene at 80°C in the presence of triethylamine provided exclusively *trans* dihydropyran **3a** in a yield of 71% and 50% e.e. (Figure 1, entry 9). Attempts to increase the enantioselectivity of the reaction by lowering the reaction temperature were thwarted by the poor solubility profile of **5**. Changing the solvent from toluene to hexanes, THF, or NMP also did not significantly impact the %e.e.

**Figure 2:**
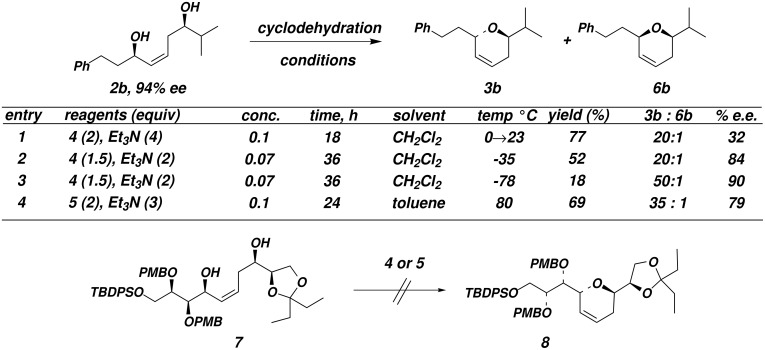
Cyclodehydration of other diol substrates

Attempts to extend these results to other systems met with limited success (Figure 2). We anticipated that the regioselectivity of dehydration of a substrate like **2b** in which the homoallylic hydroxyl is more sterically hindered than the allylic one would be improved relative to **2a**. Indeed, the cyclodehydration of **2b** with phosphorane **5** in toluene at 80°C proceeded with ca. 9 : 1 regioselectivity (entry 4), and use of the phosphonium salt **4** at -35°C also gave reasonably good results (84% e.e.). However, all attempts to dehdyrate the α,α'-oxygenated diol **7** with either **4** or **5** were unsuccessful.

**Scheme 3:**
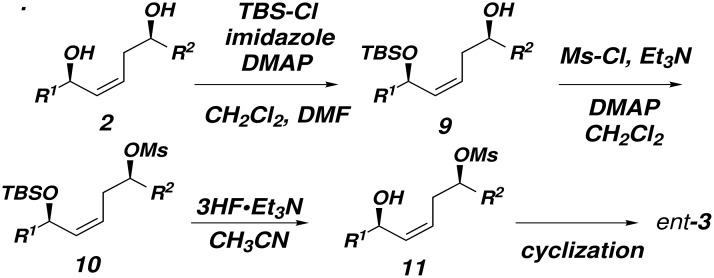


Given that the cyclodehydration reactions of diol substrates were complicated by selectivity and reactivity issues (Figure 1 and Figure 2), we turned to an alternative strategy which would not rely on chemoselectivity in the cyclodehydration step. To this end, mesylates **11** were synthesized (Scheme 3).

**Scheme 4:**
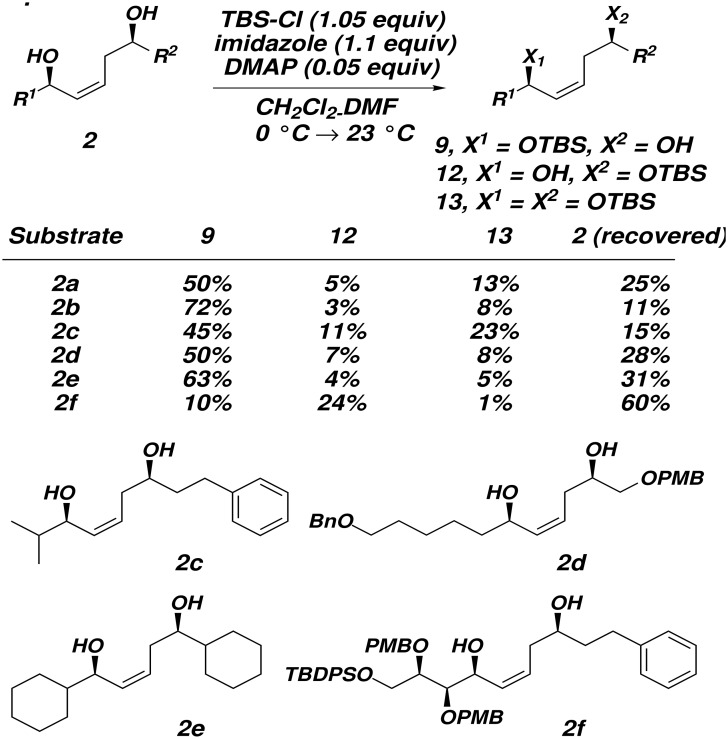


Treatment of 1,5-diols **2** with TBS-Cl and imidazole effects selective protection of the allylic alcohol (Scheme 4). The mono-TBS protected **9** is the major product in all cases except when **2f** was used as the substrate; in this case, the allylic hydroxyl is more hindered than the homoallylic hydroxyl, which is silylated preferentially. The homoallylic TBS ethers **12** and the bis-TBS ethers **13** can be conveniently recycled. The origin of the regioselectivity of the selective silylation of 1,5-diols **2** is unknown at present.

**Figure 3:**
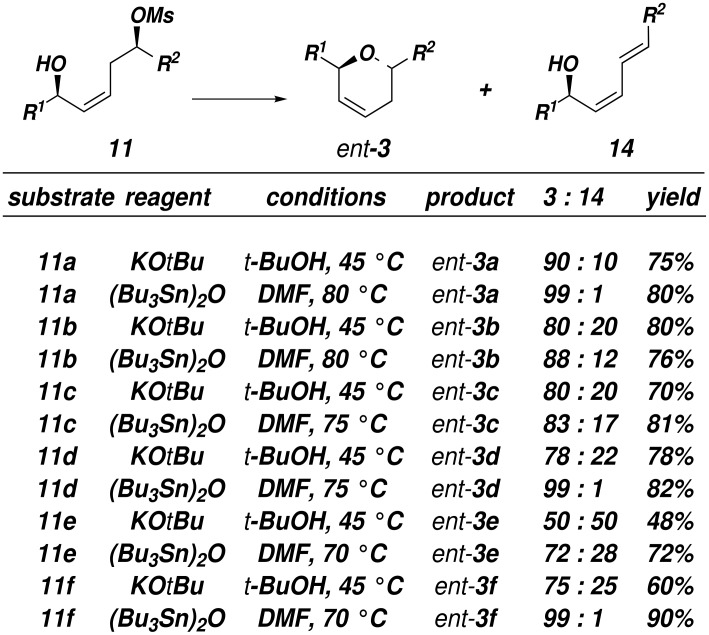
Cyclization of Hydroxymesylates **11**

Subsequent treatment of mono-TBS ethers **9** with methanesulfonyl chloride (MsCl) followed by deprotection of the TBS ether by using 3HFEt_3_N afforded mesylates **11** (80 – ≥ 95% yield). Treatment of mesylate **11a** with 1 equivalent of potassium *tert*-butoxide in *tert*-BuOH (0.01 M) between 40–50 °C provided *ent*-**3a** in 75% yield and 10:1 selectivity for the dihydropyran product and a diene resulting from an undesired E2 elimination (Figure 3, entry 1).[26–27] This result was particularly gratifying since Thomas has reported that attempts to cyclized suitably activated 1,5-diol substrates under basic conditions did not afford dihydropyran products.[28] It was necessary to carry out the reactions under dilute conditions in order to minimize elimination to diene **14**. However, increased steric demands about the mesylate (**11b**) or the allylic alcohol (**11c**) led to increased amounts of elimination, although the isolated yields of the dihydropyrans **3b** and **3c** were acceptable (Figure 3). Treatment of mesylate **11e** containing cyclohexyl groups flanking both the allylic alcohol and the mesylate gave a 1:1 mixture of *ent*-**3e** and **14e**. α-Oxygenated substrates **11d** and **11f** cyclized with 3.5:1 and 3:1 selectivity under these conditions.

It is known that tributylstannyl ethers are decent nucleophiles and are considerably less basic than oxanions.[29–30] Indeed, we found that elimination products **14** could be suppressed by treatment of alcohols **11** with (Bu_3_Sn)_2_O in benzene followed by heating the resulting tributylstannyl ethers in DMF at 80°C (Figure 3). In this way, selectivity for the formation of dihydropyran *ent*-**3** versus elimination could be increased up to 99:1 for substrates **11a**, **11d**, and **11f**. All other substrates examined also showed improved selectivity for pyran formation, and in all cases the dihydropyran *ent***-3** was obtained in at least 72% yield.

In summary, the scope of cyclodehydration reactions of (*Z*)-1,5-*syn*-diols **2** to give the the targeted dihydropyrans **3** using **4** and **5** as dehydrating reagents in a one-pot procedure is limited to substrates that lack oxygen substituents at positions adjacent to the leaving group. Acceptable enantioselectivity can be achieved by performing these cyclodehydration reactions at low temperatures. However, a much more general procedure for synthesis of the enantiomeric dihydropyrans *ent*-**3** involves the stannyl ether mediated cyclization of hydroxy mesylates **11**. This method relies on a selective silylation of the homoallylic alcohol, and represents a new route to access 2,6-*trans*-disubstituted 5,6-dihydropyrans. Application of this methodology in natural products synthesis will be reported in due course.

## Supporting Information

File 1 Experimental details.

File 2 NMR spectra for all new compounds.
