# Supplementary material for: Synthesis of 2,6-trans-disubstituted 5,6-dihydropyrans from (Z)-1,5-syn-endiols
Source: Beilstein J Org Chem. 2005 Aug 26;1:7. doi: 10.1186/1860-5397-1-7 (PMC1399455; doi:10.1186/1860-5397-1-7)
Supplement: File 1 — Experimental details. [file Beilstein_J_Org_Chem-01-07-s001.doc]

# Synthesis of 2,6-Trans-Disubstituted 5,6-Dihydropyrans from

# *(Z)*-1,5-Syn-Endiols

Eric M. Flamme^#^ and William R. Roush*^‡^

Department of Chemistry, University of Michigan, Ann Arbor, MI 48109

^‡^ Current Address: Department of Medicinal Chemistry,

Scripps Florida, Jupiter, Fl 33458

Email: [roush@scripps.edu](mailto:roush@scripps.edu)

^#^Current Address: Department of Chemistry, Princeton University

Princeton, NJ 08544

e-mail: [eflamme@princeton.edu](mailto:eflamme@princeton.edu)

**Supporting Information**

**General Details:** All reaction solvents were purified before use. Diethyl ether was dried by distillation from sodium benzophenone ketyl. Toluene was dried by distillation from sodium metal. Dichloromethane and THF were purified by passage through a solvent column composed of activated alumina (A-1).[[1]](#endnote-2) Hexanes was dried by distillation from CaH_2_. Triethylamine was dried by distillation over CaH_2_. Unless otherwise noted, all reactions were conducted under a nitrogen atmosphere using oven dried (140 °C) glassware. Nitrogen was dried by passage through a long drying column containing Drierite^TM^. Removal of solvents was accomplished on a rotary evaporator at reduced pressure. Enantiomeric excess was determined by Mosher ester analysis or Chiral HPLC as noted.[[2]](#endnote-3),[[3]](#endnote-4)

**Physical Properties and Spectroscopic Measurements:** ^1^H NMR spectra were recorded on a Varian VXR-400 spectrometer at 400 MHz or on a Varian Inova-500 spectrometer at 500 MHz. ^13^C NMR spectra were recorded on a Varian VXR-400 spectrometer at 100 MHz or a Varian Inova-500 spectrometer at 125 MHz. The proton signal of residual, non-deuterated solvent (δ 7.24 ppm for CHCl_3_) was used as an internal reference for ^1^H spectra. For ^13^C spectra, the chemical shifts are reported relative to the δ 77.0 ppm resonance of CDCl_3_ unless otherwise noted. Coupling constants are reported in Hertz (Hz).

Infrared (IR) spectra were recorded as thin films using CH_2_Cl_2_ as the solvent on a Perkin-Elmer Spectrum 1000 FTIR. Optical rotations were measured on a Rudolph Autopol III polarimeter using a quartz cell with 1 ml capacity and a 10 cm path length. Melting points were determined on a Mel-Temp II hot stage melting point apparatus and are uncorrected. Mass spectra were recorded on a VG 70-250-s spectrometer manufactured by Micromass Corp. (Manchester UK) at the University of Michigan Mass Spectrometry Laboratory.

Analytical thin layer chromatography (TLC) was performed on Kieselgel 60 F_254_ glass plates precoated with a 0.25 mm thickness of silica gel. The TLC plates were visualized with UV light and/or by staining with *p-*anisaldehyde stain (a mixture of ethanol, sulfuric acid, and *p*-anisaldehyde), unless otherwise noted. Column chromatography was generally performed according to the method of Still using Kieselgel 60 (230-400 mesh) silica gel.[[4]](#endnote-5)

HPLC purifications were performed by using an HPLC system composed of two Rainin HPXL pumps connected to a Dynamax axial compression column packed with Rainin 60 Å irregular silica gel. Samples were loaded into the system with a 2 mL Rheodyne 7125 injector and were detected by using either a Rainin Dynamax UV-C detector or a Rainin Dynamax RI-1 detector. Integration of the various signals was performed using the Reprocessing program within the Dynamax HPLC Method Manager.

Diols **2a-f** were prepared by our previously published method.[[5]](#endnote-6) Yields and characterization data for new diols are given below.

**(2*R*, 6*R*, 11-Benzyloxy-1-(*para*-methoxy-benzyloxy)-undec-4-ene-2,6-diol (2d):** Yield 77%; ee ≥76%;[[6]](#endnote-7) [α]_D_^24^ = +20.1° (c = 2.26, CHCl_3_); ^1^H NMR (500 MHz, CDCl_3_) δ 7.60-7.30 (m, 4H), 7.20-4.14 (m, 3H), 6.81-6.77 (m, 2H), 5.48-5.36 (m, 2H), 4.40 (s, 2H), 4.40-4.34 (m, 2H), 4.27-4.23 (m, 1H), 3.80-3.74 (m, 1H), 3.70 (s, 3H), 3.39-3.34 (m, 3H), 3.27 (B of ABX, *J*=7.5, 9 Hz, 1H); 2.95 (s, broad -OH, 1H), 2.69 (s, broad -OH, 1H), 2.33 (ddd, *J*=5.5, 8.5, 14.5 Hz, 1H), 2.16-2.10 (m, 1H), 1.57-1.46 (m, 3H), 1.40-1.16 (m, 5H); ^13^C NMR (125 MHz, CDCl_3_) δ 159.43, 138.73, 136.17, 130.00, 129.55, 128.43, 127.72, 127.57, 126.66, 113.97, 73.23, 73.18, 72.91, 70.43, 69.68, 67.00, 55.35, 37.16, 31.39, 29.79, 26.29, 25.33; IR (thin film) 3408, 2934, 2858, 1612, 1513, 1248, 1098 cm^-1^; HRMS (ES) calcd for C_26_H_36_O_5_Na (M+Na)^+^ 451.2460, found 451.2463 *m/z*.

**(1*R*, 5*R*, 2*E*)-1,5-dicyclohexyl-pent-2-ene-1,5-diol (2e):** Yield 70%; ee 85%; [α]_D_^24^ = +26.0° (c = 1.0, CHCl_3_); ^1^H NMR (500 MHz, CDCl_3_) δ 5.64-5.58 (m, 2H), 4.12-4.06 (m, 1H), 3.41-3.37 (m, 1H), 2.40-2.35 (m, 1H), 2.28-2.20 (m, 1H), 1.94-1.84 (m, 4H), 1.78-1.60 (m, 8H), 1.41-1.30 (m, 2H), 1.27-1.07 (m, 6H), 1.03-0.08 (m, 4H); ^13^C NMR (125 MHz, CDCl_3_, δ=77.24) δ 134.61, 128.91, 75.59, 71.87, 44.01, 42.88, 32.36, 29.46, 28.98, 28.89, 28.64, 26.74, 26.64, 26.40, 26.34, 26.23, 26.22; IR (thin film) 3350, 2923, 2852, 1449 cm^-1^; HRMS (ES) calcd for C_17_H_30_O_2_Na (M+Na)^+^ 289.2144, found 289.2134 *m/z*.

**(3*S*, 7*S*, 8*R*, 9*R*, 5*Z)*-10-(*tert*-Butyl-diphenyl-silanyloxy)-8,9-bis-(*para*-methoxy-benzyloxy)-1-phenyl-dec-5-ene-3,7-diol (2f)**: Yield 87%; dr 9:1; [α]_D_^27^ = +13.7° (c = 1.0, CHCl_3_); ^1^H NMR (500 MHz, CDCl_3_) δ 7.74-7.68 (m, 4H), 7.49-7.44 (m, 2H), 7.44-7.36 (m, 4H), 7.32-7.28 (m, 2H), 7.24-7.14 (m, 7H), 6.86-6.80 (m, 4H), 5.66-5.58 (m, 2H), 4.71 (d, *J*=11.5 Hz, 1H), 4.58-4.54 (m, 2H), 4.51 (d, *J*=11.5 Hz, 1H), 4.37 (d, *J*=11.5 Hz, 1H), 3.94-3.86 (m, 2H), 3.80 (s, 3H), 3.79 (s, 3H), 3.68-3.62 (m, 3H), 2.90 (s, broad OH, 1H), 2.80-2.74 (m, 1H), 2.68-2.60 (m, 1H), 2.33-2.39 (m, 1H), 2.3 (s, broad OH, 1H), 2.24-2.16 (m, 1H), 1.76-1.64 (m, 2H), 1.10 (s, 9H); ^13^C NMR (125 MHz, CDCl_3_) δ 159.53, 159.32, 142.30, 135.87, 135.83, 133.41, 133.30, 132.22, 130.59, 130.46, 130.08, 130.03, 130.01, 129.67, 129.52, 128.65, 128.56, 127.98, 127.96, 125.96, 114.00, 113.86, 81.49, 79.54, 74.72, 72.54, 70.26, 67.54, 62.98, 55.43, 38.89, 35.80, 32.35, 27.09, 19.40; IR (thin film) 3436, 3025, 2932, 2857, 1612, 1514, 1464, 1428, 1302, 1248, 1112, 1035 cm^-1^; HRMS (EI) calcd for C_48_H_58_O_7_SiNa (M+Na)^+^ 797.3850, found 797.3852 *m/z*. Anal. Calcd for C_48_H_58_O_7_Si: C, 74.38; H, 7.54. Found: C, 74.50; H, 7.30.

**Representative procedure for the cyclodehydration of 1,5-diols using phosphonium salt 4:** 1,5-Diol **2a** (27 mg, 0.087 mmol) was dissolved in 0.8 mL of CH_2_Cl_2_ and Et_3_N (24 μL, 0.174 mmol) was added. The reaction was cooled in an ice bath and phosphonium salt **4**[[7]](#endnote-8) (62 mg, 0.131 mmol) dissolved in 0.4 mL of CH_2_Cl_2_ was added dropwise via cannula over 10 min. The reaction was allowed to stir 1 h in the ice bath and then the bath was removed and the reaction stirred for 8 h. The solvent was removed in vacuo and the remaining residue was immediately purified by flash chromatography (95:5 Hexane-EtOAc, Hanessian) to yield 18 mg (70%) of a 20:1 mixture of dihydropyrans **3a** and **6a** as a clear oil. An analytical sample of the *trans* and *cis* isomers was prepared by HPLC (90:10, Hexanes-EtOAc). The ee of the *trans* pyran was determined by seperation of the enantiomers on an analytical Chiracel OJ HPLC column (90:10 Hexanes:Isopropylalcohol, 1ml/min).

**(*2S*, *6S*)-*2*,6-Diphenethyl-5,6-dihydro-2*H*-pyran (3a)**: ee 50%; [α]^26^_D_ = +51 (c 1.33, CHCl_3_); ^1^H NMR (500 MHz, CDCl_3_) δ 7.30-7.16 (m, 5H), 5.81-5.78 (m, 1H), 5.69-5.66 (m, 1H), 4.19-4.18 (m, 1H), 3.71 (dddd, app tt, *J* = 4.5, 4.5, 8.5, 8.5 Hz, 1H), 2.94-2.84 (m, 2H) 2.76-2.66 (m, 2H), 2.05-1.86 (m, 4H), 1.81-1.70 (m, 2H); ^13^C NMR (125 MHz, CDCl_3_, δ 77.24) δ 142.47, 142.313, 129.90, 128.75, 128.65, 128.60, 126.00, 125.98, 124.25, 71.96, 67.18, 37.43, 35.80, 32.53, 32.38, 31.11; IR (thin film) 3027, 2927, 2859, 1944(w), 1873 (w), 1803 (w), 1604 (w), 1495, 1454, 1094, 1075 cm^-1^; HRMS (EI 70ev) found [M+]: *m/z* 292.1826, calcd for C_21_H_24_O: 292.1827.

**(*2R*, *6R*)-*2*,6-Diphenethyl-5,6-dihydro-2*H*-pyran (***ent*-**3a)**: ee 40%[α]_D_^24^ = -31° (c = 0.97, CHCl_3_); ^1^H NMR (500 MHz, CDCl_3_) δ 7.30-7.14 (m, 10H), 5.81-5.76 (m, 1H), 5.70-5.66 (m, 1H), 4.20-4.15 (m, 1H), 3.71 (dddd, app tt, *J* = 4.5, 4.5, 8.5, 8.5 Hz, 1H), 2.94-2.83 (m, 2H), 2.76-2.65 (m, 2H), 2.05-1.86 (m, 4H), 1.81-1.70 (m, 2H); ^13^C NMR (125 MHz, CDCl_3_) δ 142.24, 142.08, 129.68, 128.51, 128.42, 128.36, 125.77, 125.74, 124.01, 71.71, 66.95, 37.19, 35.58, 32.29, 32.14, 30.87; IR (thin film) 3027, 2927, 1603, 1495, 1454, 1095, 1075 cm^-1^; Anal. Calcd for C_21_H_24_O: C, 86.26; H, 8.27. Found: C, 86.38; H, 8.14.

**(*2R*, *6S*)-*2*,6-Diphenethyl-5,6-dihydro-2*H*-pyran (6a)**: Analytical data for **6a** matched that reported in the literature:[[8]](#endnote-9) [α]_D_^24^ = -15° (c = 1.0, CHCl_3_); ^1^H NMR (500 MHz, CDCl_3_) δ 7.30-7.15 (m, 10H), 5.80-5.75 (m, 1H), 5.67-5.61 (m, 1H), 4.08-4.02 (m, 1H), 3.51-3.45 (m, 1H), 2.88-2.81 (m, 2H), 2.79-2.70 (m, 2H), 2.05-1.97 (m, 1H), 1.95-1.75 (m, 5H); ^13^C NMR (125 MHz, CDCl_3_) δ 142.52, 130.69, 128.80, 128.77, 128.54, 128.52, 125.92, 125.13, 74.04, 73.02, 37.85, 37.46, 31.99, 31.75, 31.63; IR (thin film) 3026, 2925, 1495, 1454, 1086 cm^-1^; HRMS (EI) calcd for C_21_H_24_O (M)^+^ 292.1827, found 292.1824 *m/z*.

The relavtive stereochemistry of **6a** was determined by an observed nOe between the hydrogens shown below.

**(2*S*, 6*R*)-*2*-Isopropyl-6-phenethyl-5,6-dihydro-2*H*-pyran (3b)**: ee 80%; [α]_D_^24^ = +105° (c = 0.8, CHCl_3_); ^1^H NMR (500 MHz, CDCl_3_) δ 7.29-7.14 (m, 5H), 5.82-5.77 (m, 1H), 5.69-5.64 (m, 1H), 4.19-4.20 (m, 1H), 3.29 (ddd appt q, *J*=9 Hz, 1H), 2.83 (ddd, *J*=14.5, 10.5, 5 Hz, 1H), 2.71-2.64 (m, 1H), 2.0-1.9 (m, 3H), 1.73-1.63 (m, 2H), 1.04 (d, *J*=6.5 Hz, 3H), 0.92 (d, *J*=7 Hz, 3H); ^13^C NMR (100 MHz, CDCl_3_) δ 142.27, 129.69, 128.52, 128.34, 125.72, 124.34, 72.74, 72.31, 35.42, 32.89, 32.36, 28.52, 19.15, 18.66; IR (thin film) 3085 (w), 3063 (w), 3028, 2957, 2927, 2873, 1940 (w), 1658 (w), 1604, 1496, 1470, 1454, 1194, 1092, 1075 cm^-1^; HRMS (EI) calcd for C_16_H_22_O_2_ (M)^+^ 230.1670, found 230.1669 *m/z*.

**(2*R*, 6*S*)-*2*-Isopropyl-6-phenethyl-5,6-dihydro-2*H*-pyran** **(***ent*-**3b)**: ee 94%; [α]_D_^26^ = -78.0° (c = 0.8, CHCl_3_); ^1^H NMR (500 MHz, CDCl_3_) δ 7.32-7.18 (m, 5H), 5.85-5.81 (m, 1H), 5.72-5.68 (m, 1H), 4.21-4.16 (m, 1H), 3.36-3.30 (m, 1H), 2.87 (ddd, *J*=5, 10.5, 14.5 Hz, 1H), 2.71 (ddd, *J*=7, 10, 14 Hz, 1H), 2.04-1.94 (m, 3H), 1.76-1.68 (m, 2H), 1.08 (d, *J*=6.5 Hz, 3H), 0.96 (d, *J*=7 Hz, 3H); ^13^C NMR (125 MHz, CDCl_3_) δ 142.25, 129.68, 128.52, 128.34, 125.72, 124.33, 72.46, 72.31, 35.41, 32.89, 32.35, 28.52, 19.16, 18.66; IR (thin film) 3027, 2955, 2928, 1495, 1455, 1091, 1076 cm^-1^; HRMS (EI) calcd for C_16_H_22_O (M)^+^ 230.1671, found 230.1671 *m/z*.

**General procedure for cyclodehydration of 1,5-diols with 4** **at low temperature**: 1,5-Diol **2b** (37 mg, 0.15 mmol) was dissolved in 1.5 mL of CH_2_Cl_2_ and Et_3_N (41 μL, 0.30 mmol) was added. The reaction was cooled to -78 °C in an acetone-dry ice bath. Phosphonium salt **4** dissolved in 1 mL of CH_2_Cl_2_ was added to the reaction dropwise over 20 min. The reaction was stirred for 36 h at -78 °C. The reaction was quenched at -78 °C by the additional of 1 mL of MeOH. The reaction was diluted with 15 mL of CH_2_Cl_2_, washed 2 x sat. NaHCO_3_, dried over Na_2_SO_4_, and filtered. Removal of the solvent in vacuo and purification by flash chromatography (95:5 Hexanes-EtOAc, Hanessian) provided 6 mg (18%) of a 20:1 mixture of pyrans **3b** and **6b** as a clear oil. An analytical sample was prepared by HPLC (97:3 Hexanes-EtOAc). The ee of the *trans* pyran was determined by seperation of the enantiomers on an analytical Chiracel OD HPLC column (99.25:0.75 Hexanes:Isopropylalcohol, 1ml/min).

**General procedure for cyclodehydration of 1,5-diols with phosphorane 5:** 1,5-Diol **2b** (0.038 mg, 0.12 mmol) was dissolved in 1 ml of hexanes under argon. Triethyl amine (69 μl, 0.429 mmol) was added followed by phosphorane **5[[9]](#endnote-10)** (0.25 mmol; as a 0.5 M sol in toluene). The reaction was stirred under argon and heated to 80 °C for 24 h. The reaction was cooled, diluted with hexanes and washed 1 x sat. brine. The organic layer was dried over Na_2_SO_4_ and purified by column chromatography (95:5 Hexanes-EtOAc) to yield 19 mg (60%) of pyran **3b** as a clear oil.

**Representative procedure for the selective TBS protection of 1,5-diols: (3*R*, 7*S*, 4*Z*)-7-(*tert*-Butyl-dimethyl-silanyloxy)-1,9-diphenyl-non-5-en-3-ol (9a):** Diol **2a** (90 mg, 0.29 mmol) was dissolved in 1.6 mL of a 50:50 solution of CH_2_Cl_2_–DMF. Imidazole (20 mg, 0.30 mmol) and DMAP (2mg, 0.015 mmol) were added to the reaction followed by TBSCl (45 mg, 0.30 mmol). The reaction was stirred for 18 h at ambient temperature and quenched with H_2_O. The reaction was diluted with 90:10 Hexanes–EtOAc and the combined organices were washed 1 x sat. NaHCO_3_, 1 x brine, dried over Na_2_SO_4_, and filtered. The organics were removed in vacuo and the crude oil was purified by column chromatography (85:15, Hexanes-EtOAc) to afford 64 mg (50%) of mono-protected alcohol **9a** as a clear oil. Additionally, Bis-protected product **13a** (13%), regio isomeric mono-protected product **12a** (5%), and reclaimed starting material (25%) were also isolated. Partial data for **9a**: ^1^H NMR (500 MHz, CDCl_3_) δ 7.27-7.17 (m, 10H), 5.62-5.58 (m, 1H), 5.42-5.39 (m, 1H), 4.45-4.41 (m, 1H), 3.70-3.62 (m, 1H), 2.82-2.59 (m, 4H), 2.28-2.19 (m, 2H), 1.92-1.70 (m, 4H), 1.60 (s, -OH, broad, 1H), 0.92 (s, 9H), 0.04 (s, 3H), 0.01 (s, 3H)

**(2*R*, 6*R*, 4*E*)-11-Benzyloxy-6-(*tert*-butyl-dimethyl-silanyloxy)-1-(*para*-methoxy-benzyloxy)-undec-4-en-2-ol (9d):** Partial data: ^1^H NMR (500 MHz, CDCl_3_) δ 7.32-7.30 (m, 3H), 7.28-7.22 (m, 4H), 6.88-6.85 (m, 2H), 5.47-5.43 (m, 1H), 5.35-5.29 (m, 1H), 4.47 (s, 2H), 4.46 (s, 2H), 4.36-4.31 (s, 1H), 3.82-3.79 (m, 1H), 3.78 (s, 3H), 3.46 (A of ABX, *J* = 3, 9.5 Hz, 1H); 3.43 (t, *J* = 6.5 Hz, 2H), 3.29 (B of ABX, *J* = 8, 9.5 Hz, 1H), 2.32 (s, -OH, broad, 1H), 2.26-2.16 (m, 2H), 1.62-1.46 (m, 4H), 1.39-1.23 (m, 4H), 0.84 (s, 9H), 0.00 (s, 3H), -0.02 (s, 3H).

**(1*R*, 5*R*, 2*E*)-1-(*tert*-Butyl-dimethyl-silanyloxy)-1,5-dicyclohexyl-pent-2-en-5-ol (9e)**: Partial data: ^1^H NMR (500 MHz, CDCl_3_) δ 5.52-5.45 (m, 2H), 4.15-4.08 (m, 1H), 3.42-3.39 (m, 1H), 2.38-2.30 (m, 1H), 2.18-2.10 (m, 1H), 1.92-1.60 (m, 12H), 1.41-0.92 (m, 11), 0.90 (s, 9H), 0.04 (s, 3H), 0.01 (s, 3H).

**Representative procedure for the synthesis of mesylate substrates 11a-f: (3*R*, 7*R*, 4*Z*)-1-phenyl-7-methylsulfonyloxy-8-methylnon-4ene-3-ol (11b)**: Alcohol **9b** (100mg, 0.273 mmol) was dissolved in 3 mL of CH_2_Cl_2_. To the solution was added Et_3_N (0.19 mL, 1.36 mmol) and DMAP (3.5 mg, 0.027 mmol). The reaction was cooled in an ice bath and methanesulfonyl chloride (0.22 mL, 2.73 mmol) was added dropwise and the reaction stirred 1 h. TLC analysis (95:5, CH_2_Cl_2_-Et_2_O) showed the reaction was complete. The reaction was diluted with CH_2_Cl_2_ and washed with NH_4_Cl, sat. NaHCO_3_ (2X), and brine. The organics were dried over Na_2_SO_4_, filtered, and the solvent removed in vacuo. The crude oil was purified by column chromatography (80:20 hexanes-EtOAc, Hanessian stain) to afford 116 mg (95%) of mesylate **10b** as a colorless oil. The oil **10b** (116 mg, 0.262 mmol) was dissolved in 2 mL of CH_3_CN and 3HF•Et_3_N (0.64 mL, 3.93 mmol) was added. The reaction was stirred at ambient temperature for 18 h. The reaction was diluted with CH_2_Cl_2_ and with sat. NaHCO_3_ (2x). The organics were dried over Na_2_SO_4_, filtered, and the solvent removed in vacuo. The crude oil was purified by column chromatography (70:30, hexanes:EtOAc, Hanessian stain) to afford 83 mg (96%) of **11b** as a colorless oil: [α]_D_^24^ = +36.2° (c = 1.05, CHCl_3_); ^1^H NMR (500 MHz, CDCl_3_) δ 7.32-7.27 (m, 2H), 7.23-7.18 (m, 3H), 5.65 (dd, *J*=9, 11 Hz, 1H), 5.55 (ddd, app dt, *J*=8, 8, 11 Hz, 1H), 4.57-4.54 (m, 1H), 4.45-4.41 (m, 1H), 3.00 (s, 3H), 2.78-2.66 (m, 2H), 2.54 (dddd, *J*=1.5, 5.8, 15 Hz, 1H), 2.45 (dddd, *J*=1.5, 7, 7, 15, 1H), 2.10-1.90 (m, 3H), 1.82-1.75 (m, 1H), 0.99 (d, *J*=6 Hz, 3H), 0.97 (d, *J*=6 Hz, 3H); ^13^C NMR (125 MHz, CDCl_3_, δ = 77.24) δ 141.92, 136.29, 128.60, 126.07, 125.64, 87.77, 66.98, 38.93, 38.86, 31.78, 31.34, 29.91, 18.43, 17.94; IR (thin film) 3534, 2967, 2936, 1335, 1172, 906 cm^-1^; HRMS (ES) calcd for C_17_H_26_O_4_SNa (M+Na)^+^ 349.1450, found 349.1454 *m/z*.

**(3*R*, 7*S*, 4*Z*)-1,9-diphenyl-7-methansulfonyloxynon-4-ene-3-ol (11a)**: [α]_D_^26^ = +17° (c = 1.2, CHCl_3_); ^1^H NMR (500 MHz, CDCl_3_) δ 7.31-7.25 (m, 4H), 7.21-7.15 (m, 6H), 5.66-5.61 (m, 1H), 5.55-5.49 (m, 1H), 4.78-4.73 (m, 1H), 4.41-4.36 (m, 1H), 2.96 (s, 3H), 2.77-2.57 (m, 5H), 2.46-2.40 (m, 1H), 2.09-2.01 (m, 1H), 1.98-1.88(m, 3H), 1.78-1.70 (m, 1H); ^13^C NMR (125 MHz, CDCl_3_) δ 141.63, 140.45, 136.39, 128.56, 128.37, 128.36, 128.29, 126.24, 125.85, 124.69, 81.87, 66.73, 38.68, 38.62, 35.53, 32.74, 31.55, 31.40; IR (thin film) 3537, 3026, 2933, 1496, 1454, 1334, 1171 cm^-1^; HRMS (ES) calcd for C_22_H_28_O_4_SNa (M+Na)^+^ 411.1606, found 411.1607 *m/z*.

**(3*S*, 7*R*, 5*Z*)-1-phenyl-3-methansulfonyloxy-8-methylnon-5-ene-7-ol (11c)**: [α]_D_^27^ = -5.8° (c = 1.04, CHCl_3_); ^1^H NMR (500 MHz, CDCl_3_) δ 7.30-7.24 (m, 2H), 7.21-7.16 (m, 3H), 5.62-5.50 (m, 2H), 4.80-4.74 (m, 2H), 4.05 (dd, *J*=6.5, 8 Hz, 1H), 2.98 (s, 3H), 2.80-2.74 (m, 1H), 2.71-2.63 (m, 2H), 2.53-2.46 (m, 1H), 2.10-2.03 (m, 1H), 2.02-1.94 (m, 1H), 1.67 (dqq app octet, *J*=6.5, 6.5, 6.5 Hz, 1H), 0.94 (d, *J*=6.5 Hz, 3H), 0.84 (d, *J*=6.5 Hz, 3H); ^13^C NMR (125 MHz, CDCl_3_) δ 140.52, 134.93, 128.53, 128.30, 126.20, 125.24, 81.95, 72.33, 38.63, 35.53, 34.01, 32.88, 31.37, 18.06; IR (thin film) 3538, 2958, 2872, 1455, 1334, 1171, 907 cm^-1^; HRMS (ES) calcd for C_17_H_26_O_4_SNa (M+Na)^+^ 349.1450, found 349.1448 *m/z*.

**(2*R*, 6*R*, 4*E*)-1-*para*-methoxybenzyloxy-2-methansulfonyloxy-11-benzyloxy-undec-5-ene-7-ol (11d)**: [α]_D_^24^ = +7.5° (c = 1.17, CHCl_3_); ^1^H NMR (500 MHz, CDCl_3_) δ 7.26-7.22 (m, 3H), 7.20-7.12 (m, 4H), 6.81-6.77 (m, 2H), 5.51-5.46 (m, 1H), 5.39-5.33 (m, 1H), 4.74-4.70 (m, 1H), 4.43-4.36 (m, 4H), 4.30-4.24 (m, 1H), 3.71 (s, 3H), 3.54-3.46 (m, 2H), 3.37 (t, *J*=13 Hz, 2H), 2.9 (s, 3H), 2.59-2.51 (m, 1H), 2.39-2.32 (m, 1H), 1.72 (s, broad OH, 1H), 1.56-1.46 (m, 3H), 1.38-1.15 (m, 5H); ^13^C NMR (125 MHz, CDCl_3_) δ 159.43, 138.64, 136.96, 129.46, 129.28, 128.31, 127.50, 127.45, 124.13, 113.88, 81.19, 73.14, 72.82, 70.40, 70.29, 67.23, 55.24, 38.59, 37.11, 30.09, 29.65, 26.14, 25.11; IR (thin film) 3435, 2935, 2859, 1613, 1514, 1353, 1248, 1173, 1094 cm^-1^; HRMS (ES) calcd for C_27_H_38_O_7_SNa (M+Na)^+^ 529.2236, found 529.2244 *m/z*.

**(1*R*, 5*R*, 3*E*)-1,5-dicyclohexyl-1-methanesulfonyloxypenta-3-ene-5-ol (11e)**: [α]_D_^24^ = +13.9° (c = 1.3, CHCl_3_); ^1^H NMR (500 MHz, CDCl_3_) δ 5.56-5.48 (m, 2H), 4.50 (ddd, app q, *J* = 6, 6, 6 Hz, 1H), 4.06-4.03 (m, 1H), 2.96 (s, 3H), 2.64-2.57 (m, 1H), 2.45-2.38 (m, 1H), 1.90-1.84 (m, 2H), 1.80-1.56 (m, 10 H), 1.34-1.28 (m, 1H), 1.24-0.84 (m, 10 H); ^13^C NMR (125 MHz, CDCl_3_, δ = 77.24) δ 135.06, 125.83, 87.27, 71.79, 43.93, 40.61, 38.79, 30.00, 28.94, 28.86, 28.72, 28.31, 26.64, 26.24, 26.23, 26.09, 26.02, 25.93; IR (thin film) 3542, 2927, 2853, 1450, 1334, 1172 cm^-1^; HRMS (ES) calcd for C_18_H_32_O_4_SNa (M+Na)^+^ 367.1919, found 367.1915 *m/z*.

**Synthesis of** **mesylate (11f)**: Although **11f** can be synthesized following Scheme 3 of the paper, a more efficient synthesis is as follows: Alcohol **SI-1** (20 mg, 0.024 mmol) was dissolved in 0.5 mL of CH_2_Cl_2_. To the solution was added Et_3_N (17 μl, 0.12 mmol) and DMAP (0.3 mg, 2.4 μmol). The reaction was cooled in an ice bath and methanesulfonyl chloride (18 μl, 0.24 mmol) was added dropwise and the reaction stirred 1 h. TLC analysis (95:5, CH_2_Cl_2_-Et_2_O) showed the reaction was complete. The reaction was diluted with CH_2_Cl_2_ and washed with NH_4_Cl, sat. NaHCO_3_ (2x), and brine. The organics were dried over Na_2_SO_4_, filtered, and the solvent removed in vacuo. The crude oil was purified by column chromatography (80:20 hexanes-EtOAc, Hanessian stain) to afford 18 mg (81%) of mesylate **SI-4** as a colorless oil. The oil **SI-4** (18 mg, 0.019 mmol) was dissolved in 1 mL of MeOH and K_2_CO_3_ (20mg, 0.20 mmol) was added. The reaction was stirred vigorously for 10 min and then diluted with a 80:20 solution of hexanes–EtOAc. The organics were washed 1 x NaHCO_3_, 1 x brine, dried over Na_2_SO_4_, and filtered. The solvent was removed in vacuo and the crude oil was purified by column chromatography (60:40, hexanes-EtOAc, Hanessian stain) to afford 15 mg (97%) of mesylate **11f** as a colorless oil: [α]_D_^24^ = +18.3° (c = 1.20, CHCl_3_); ^1^H NMR (500 MHz, CDCl_3_) δ 7.68-7.63 (m, 4H), 7.44-7.32 (m 6H), 7.28-7.23 (m, 2H), 7.18-7.10 (m, 7H), 6.82-6.76 (m, 4H), 5.66-5.60 (m, 1H), 5.52-5.46 (m, 1H), 4.73-4.68 (m, 1H), 4.62 (A of ABq, *J*=11 Hz, 1H), 4.52 (dd, *J*=8.5, 13 Hz, 1H), 4.49 (Bof ABq, *J*=11 Hz, 1H), 4.46 (A of ABq, *J*=11 Hz, 1H), 4.32 (B of ABq, *J*=11 Hz, 1H), 3.88-3.81 (m, 2H), 3.77 (s, 3H), 3.75 (s, 3H), 3.62-3.57 (m, 2H), 2.90 (s, 3H), 2.72-2.54 (m, 3H), 2.43-2.36 (m, 1H), 2.27 (s, broad, OH, 1H), 2.00-1.86 (m, 2H), 1.04 (s, 9H); ^13^C NMR (125 MHz, CDCl_3_, δ = 77.24) δ 159.56, 159.36, 140.90, 135.91, 135.85, 133.42, 133.31, 130.65, 130.48, 130.07, 130.05, 130.02, 129.53, 128.74, 128.59, 128.02, 127.98, 126.55, 126.37, 114.01, 113.92, 82.28, 81.38, 79.81, 74.59, 72.65, 67.37, 63.10, 55.48, 38.82, 36.02, 33.18, 31.42, 27.11, 19.41; IR (thin film) 3543, 2932, 2857, 1612, 1513, 1355, 1248, 1173, 1112, 1034 cm^-1^; HRMS (ES) calcd for C_49_H_60_O_9_SSiNa (M+Na)^+^ 875.3625, found 875.3622 *m/z*.

**Representavtive procedure for the synthesis of dihydropyrans from hydroxy mesylates using potassium *tert-*butoxide**: Mesylate **11a** (35 mg, 0.90 mmol) was dissolved in 9 mL of dry *tert*-BuOH (distilled from CaH_2_). The solution was heated to 45° C and 90 μL of *t*-BuOK (1M in *tert-*BuOH; made by adding solid *t-*BuOK to dry *tert*-BuOH) was added to the reaction. The reaction was heated 24 h and then cooled. The solvent was removed in vacuo (Note: Care should be taken as *tert*-BuOH can freeze and/or bump during in vacuo removal) and the remaining slurry diluted with a mixture of 80:20 Hexanes-EtOAc and sat. NaHCO_3_ solution. The ogranics were washed with NaHCO_3_, brine, dried over Na_2_SO_4_, and filtered. The crude oil was purified by column chromatography (90:10 hexanes:EtOAc) to afford 20 mg (75%) of dihydropyran *ent-***3a** as a clear oil.

**(2*R*, 6*R*)-2-Isopropyl-6-phenethyl-5,6-dihydro-2*H*-pyran (*ent*-3c)**: [α]_D_^24^ = -61.1° (c = 0.7, CHCl_3_); ^1^H NMR (500 MHz, CDCl_3_) δ 7.28-7.24 (m, 2H), 7.21-7.14 (m, 3H), 5.83-5.74 (m, 2H), 3.74-3.64 (m, 2H), 2.87 (ddd, *J*=5.5, 10.5, 14 Hz, 1H), 2.64 (ddd, *J*=7, 10, 14 Hz, 1H), 2.06-2.00 (m, 1H), 1.94-1.80 (m, 3H), 1.76-1.69 (m, 1H), 1.02 (d, *J*=6.5 Hz, 3H), 0.92 (d, *J*=7 Hz, 3H); ^13^C NMR (125 MHz, CDCl_3_) δ 142.37, 128.39, 128.35, 128.31, 125.69, 124.23, 77.56, 67.93, 37.12, 32.31, 32.23, 30.63, 19.53, 18.89; IR (thin film) 3028, 2956, 2927, 1454, 1074 cm^-1^; HRMS (EI) calcd for C_16_H_22_ O (M)^+^ 230.1671, found 230.1674 *m/z*.

**(2*R*, 6*S*)-2-(5-Benzyloxy-pentyl)-6-(*para*-methoxy-benzyloxymethyl)-5,6-dihydro-2*H*-pyran (*ent*-3d)**: [α]_D_^25^ = -55.1° (c = 1.1, CHCl_3_); ^1^H NMR (500 MHz, CDCl_3_) δ 7.38-7.33 (m, 4H), 7.32-7.27 (m, 3H), 6.91-6.87 (m, 2H), 5.82-5.77 (m, 1H), 5.74-5.69 (m, 1H), 4.55 (A of ABq *J*=12 Hz, 1H), 4.51 (B of ABq, *J*=12 Hz, 1H), 4.51 (s, 2H), 4.19-4.14 (m, 1H), 3.95-3.89 (m, 1H), 3.81 (s, 3H), 3.54 (A of ABX, *J*=6, 10 Hz, 1H), 3.48 (t, *J*=6.5 Hz, 2H), 3.47 (B of ABX, *J*=5, 10 Hz, 1H), 2.08-1.94 (m, 2H), 1.71-1.61 (m, 3H), 1.57-1.48 (m, 5H); ^13^C NMR (125 MHz, CDCl_3_, δ = 77.24) δ 159.33, 138.90, 130.71, 130.13, 129.46, 128.55, 127.83, 127.68, 123.50, 113.92, 73.20, 73.07, 72.81, 72.68, 70.61, 67.07, 55.46, 34.19, 29.96, 27.47, 26.37, 25.95; IR (thin film) 2932, 2857, 1613, 1513, 1247, 1100 cm^-1^; HRMS (ES) calcd for C_26_H_34_O_4_Na (M+Na)^+^ 433.2355, found 433.2349 *m/z*. Anal. Calcd for C_26_H_34_O_4_: C, 76.06; H, 8.35. Found: C, 76.30; H, 8.55.

**(2*R*, 6*S*)-2,6-Dicyclohexyl-5,6-dihydro-2*H*-pyran (*ent*-3e)**: [α]_D_^29^ = -82.7° (c = 1.0, CHCl_3_); ^1^H NMR (500 MHz, CDCl_3_) δ 5.83-5.77 (m, 2H), 3.73 (dd, *J*=1, 8.5 Hz, 1H), 3.30 (ddd, *J*=4, 8, 8 Hz, 1H), 2.08-1.88 (m, 4H), 1.76-1.60 (m, 8H), 1.59-1.50 (m, 1H), 1.38-1.30 (m, 1H), 1.26-1.08 (m, 6H), 1.20-0.90 (m, 4H); ^13^C NMR (125 MHz, CDCl_3_) δ 128.42, 124.33, 77.45, 72.33, 42.64, 41.82, 29.92, 29.64, 29.31, 28.79, 28.37, 26.62, 26.52, 26.18, 26.02, 25.98; IR (thin film) 2922, 2851, 1449, 1082 cm^-1^; HRMS (EI) calcd for C_17_H_28_O (M)^+^ 248.2140, found 248.2147 *m/z*. Anal. Calcd for C_17_H_28_O: C, 82.20; H, 11.36. Found: C, 82.22; H, 11.15.

**Dihydropyran *ent*-3f**: [α]_D_^25^ = -66.5° (c = 1.08, CHCl_3_); ^1^H NMR (500 MHz, CDCl_3_) δ 7.68-7.63 (m, 4H), 7.41-7.36 (m, 2H), 7.34-7.28 (m, 4H), 7.22-7.08 (m, 9H), 6.80-6.75 (m, 4H), 5.86-5.81 (m, 1H), 5.52-5.48 (m, 1H), 4.61 (A of ABq, *J*=11 Hz, 1H), 4.60 (A of ABq, *J*=11.5 Hz, 1H), 4.56 (B of ABq, *J*=11 Hz, 1H), 4.44-4.41 (m, 1H), 4.41 (B of ABq, *J*=11.5 Hz, 1H), 3.98-3.92 (m, 1H), 3.91-3.89 (m, 2H), 3.76 (s, 6H), 3.74-3.68 (m, 2H), 2.67 (ddd, *J*=5.5, 10.5, 14 Hz, 1H), 2.53 (ddd, *J*=6, 10.5, 14 Hz, 1H), 2.08-2.10 (m, 1H), 1.92-1.78 (m, 2H), 1.70-1.62 (m, 2H), 1.03 (s, 9H); ^13^C NMR (125 MHz, CDCl_3_, δ = 77.24) δ 159.17, 159.14, 142.50, 135.90, 135.86, 133.77, 133.68, 131.38, 131.21, 129.84, 129.80, 129.73, 129.60, 128.58, 128.48, 127.89, 127.84, 127.03, 125.85, 125.50, 113.78, 113.70, 80.51, 74.29, 72.90, 72.70, 69.02, 64.02, 55.46, 55.45, 37.51, 32.20, 30.44, 27.13, 19.46; IR (thin film) 2930, 2856, 1612, 1513, 1248, 1112, 1036 cm^-1^; HRMS (ES) calcd for C_48_H_56_O_6_SiNa (M+Na)^+^ 779.3744, found 779.3759 *m/z*.

**Representative procedure for the formation of dihyrdropyrans *ent*-3a-f using bis(tributyltin) oxide:** Mesylate **11a** (10 mg, 25 μmol) was dissolved in 1.5 mL of benzene and a condensor was placed on top of the reaction flask. Bis(tributyltin)oxide (6 μL, 13 μmol) was added and the reaction was heated at 75 °C for 4 h. The reaction was cooled to 40 °C at which time the condensor was removed and replaced with a short path distillation apparatus. The benzene was removed in vacuo with the distillation apparatus and the residual oil was place under high vacuum for 1 h. The flask was removed from the vacuum line and 3 mL of DMF was added and a condensor placed on top of the reaction flask. The reaction was heated to 75 °C and stirred for 30 h. The reaction was cooled and diluted with H_2_O and a mixute of 90:10 hexanes–EtOAc. The organics were washed with H_2_O and the combined aqueous layers were back extracted with 90:10 hexanes:EtOAc. The combined organics were washed 1 x brine, dried over Na_2_SO_4_, and filtered. The solvents were removed in vacuo and the crude oil purified by flash chromatography (90:10, hexanes-EtOAc) to give 6 mg (80%) of dihydropyran *ent*-**3a** as a clear oil.

**References**

1. Pangborn, A. D.; Giardello, M. A.; Grubbs, R. H.; Rosen, R. K.; timmers, F. J. *Organometallics* **1996**, *15*, 1518. [↑](#endnote-ref-2)
2. Dale, J. A.; Mosher, H. S. *J. Am. Chem. Soc.* **1973**, *95*, 512. [↑](#endnote-ref-3)
3. Ohtani, I.; Kusumi, T.; Kashman, Y.; Kakisawa, H. *J. Am. Chem. Soc.* **1991**, *113*, 4092. [↑](#endnote-ref-4)
4. Still, W. C.; Kahn, M.; Mitra, A. *J. Org. Chem.* **1978**, *43*, 2923. [↑](#endnote-ref-5)
5. Flamme, E. M.; Roush, W. R. *J. Am. Chem. Soc.* **2002**, *124*, 13644. [↑](#endnote-ref-6)
6. Enantiomeric excess could only be estimated due to overlapping signals in the ^1^H NMR spectra of the bis—Mosher ester. [↑](#endnote-ref-7)
7. Phillips, D. I.; Szele, I.; Westheimer, F. H. *J. Am. Chem. Soc.* **1976**, *98*, 184. [↑](#endnote-ref-8)
8. Dilley, G. J. *Ph. D. Thesis* **2000**, University of Michigan. [↑](#endnote-ref-9)
9. Kelly, J. W.; Robinson, P. L.; Evans, Jr., S. A. *J. Org. Chem*. **1986**, *51,* 4473. [↑](#endnote-ref-10)
