# Supplementary material for: Synthesis of 2,6-trans-disubstituted 5,6-dihydropyrans from (Z)-1,5-syn-endiols
Source: Beilstein J Org Chem. 2005 Aug 26;1:7. doi: 10.1186/1860-5397-1-7 (PMC1399455; doi:10.1186/1860-5397-1-7)
Supplement: File 2 — NMR spectra for all new compounds. [file Beilstein_J_Org_Chem-01-07-s002.pdf]

Synthesis of 2,6-Trans-Disubstituted 5,6-Dihydropyrans from *(Z)*-1,5-Syn-Endiols

Eric M. Flamme[#] and William R. Roush*[‡]

Department of Chemistry, University of Michigan, Ann Arbor, MI 48109

[‡] Current Address: Department of Medicinal Chemistry,
Scripps Florida, Jupiter, Fl 33458
Email: roush@scripps.edu

[#]Current Address: Department of Chemistry, Princeton University
Princeton, NJ 08544
e-mail: eflamme@princeton.edu

Supporting Information: Copies of ^{1}H NMR Spectra

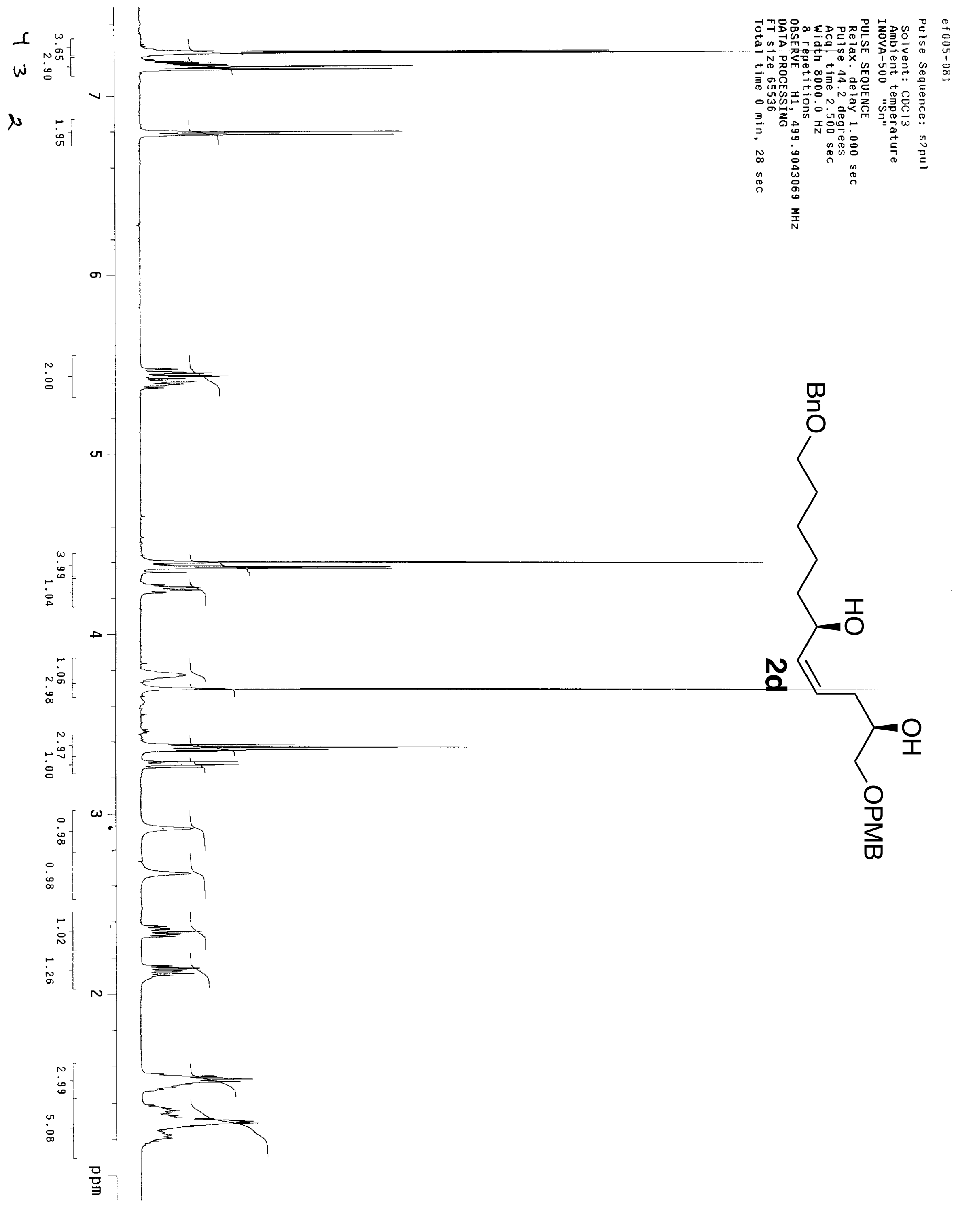
ef005-081
Pulse Sequence: s2pul
Solvent: CDCl3
Ambient temperature
INOVA-500 "Sn"
PULSE SEQUENCE
Relax. delay 1.000 sec
Pulse 44.2 degrees
Acq. time 2.500 sec
Width 8000.0 Hz
8 repetitions
OBSERVE H1, 499.9043069 MHz
DATA PROCESSING
FT size 65536
Total time 0 min, 28 sec
BnO
HO
OH
OPMB
2d
ppm

ef005-075

Pulse Sequence: s2pul

Solvent: CDCl3
Ambient temperature
INOVA-500 "Sn"

PULSE SEQUENCE
Relax. delay 1.000 sec
Pulse 44.2 degrees
Acq. time 2.500 sec
Width 8000.0 Hz
8 repetitions
OBSERVE H1, 499.9042708 MHz
DATA PROCESSING
FT size 65536
Total time 0 min, 28 sec

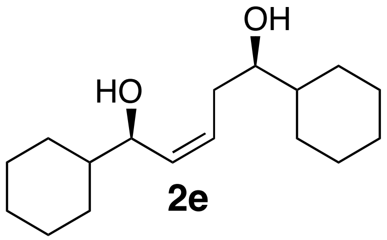

2e

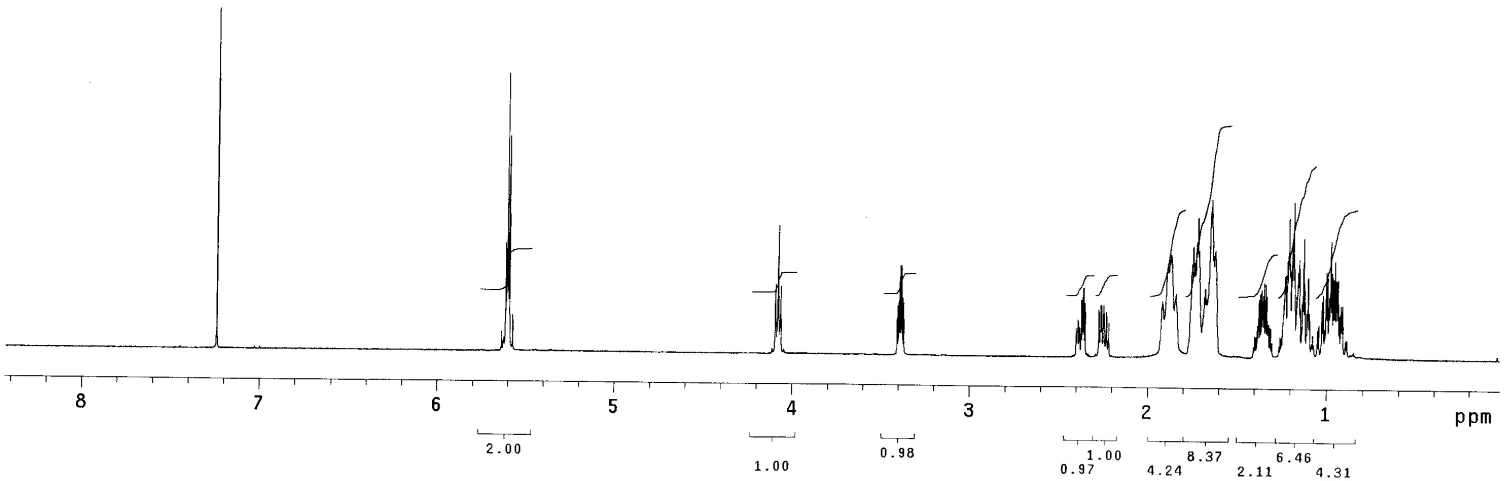

1

Pulse Sequence: s2pul
Solvent: CDCl3
Ambient temperature
INOVA-500 "Sn"
PULSE SEQUENCE
Relax. delay 1.000 sec
Pulse 44.6 degrees
Acq. time 2.500 sec
Width 8000.0 Hz
32 repetitions
OBSERVE H1, 499.9042537 MHz
DATA PROCESSING
FT size 65536
Total time 1 min, 52 sec

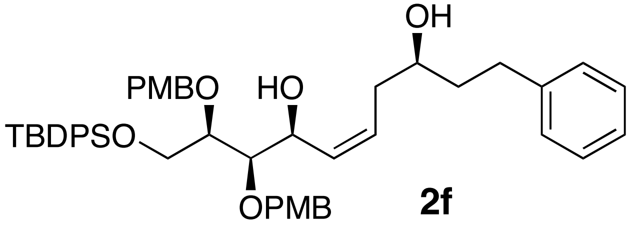

2f

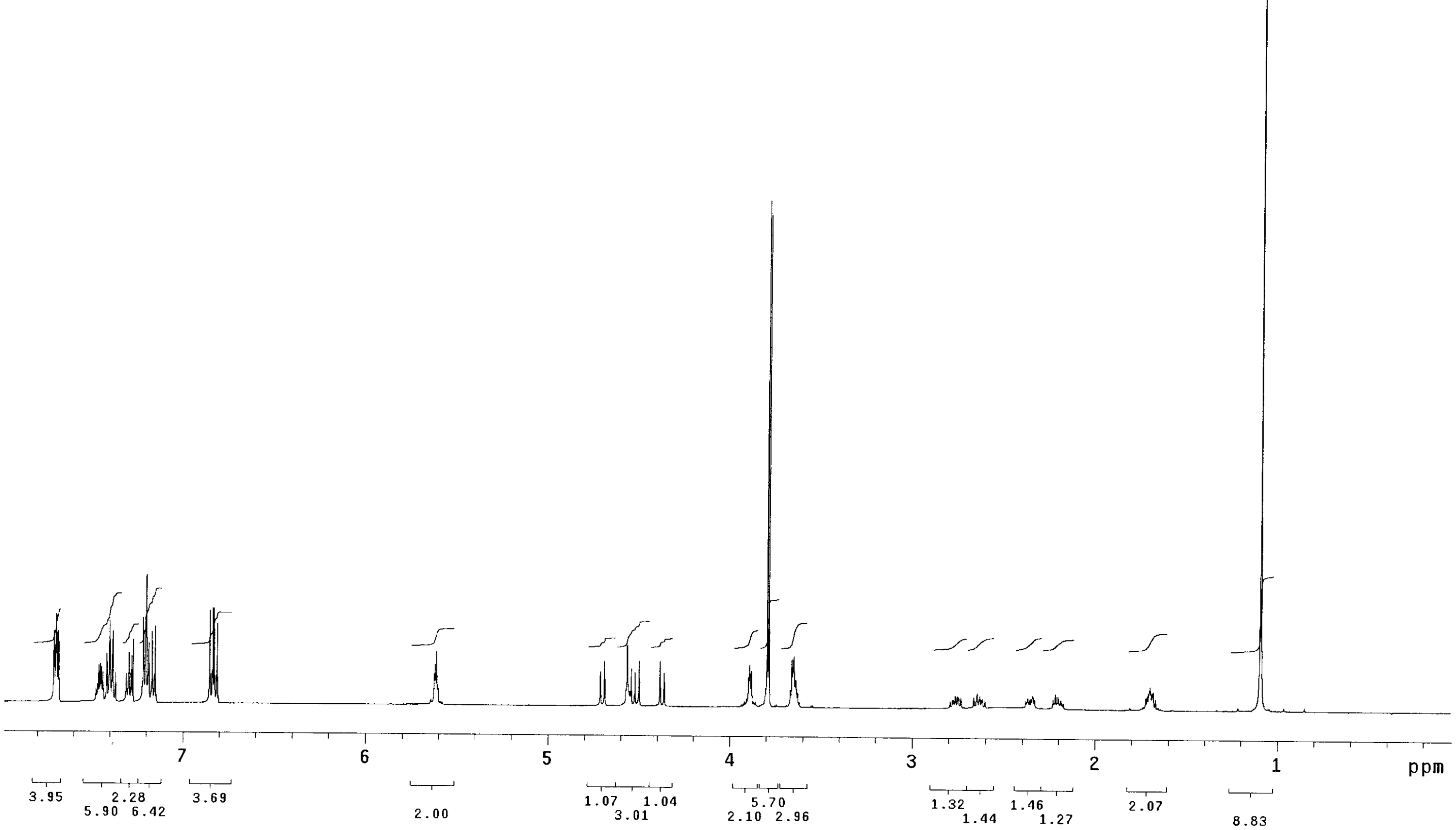

ef002-198

Pulse Sequence: s2pul

Solvent: CDCl3
Ambient temperature
INOVA-500 "Sn.Chem.LSA.UMich.Edu"

PULSE SEQUENCE
Relax. delay 1.000 sec
Pulse 40.0 degrees
Acq. time 2.500 sec
Width 8000.0 Hz
8 repetitions
OBSERVE H1, 499.9042537 MHz
DATA PROCESSING
FT size 65536
Total time 0 min, 28 sec

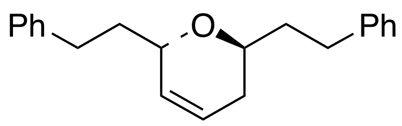

3a

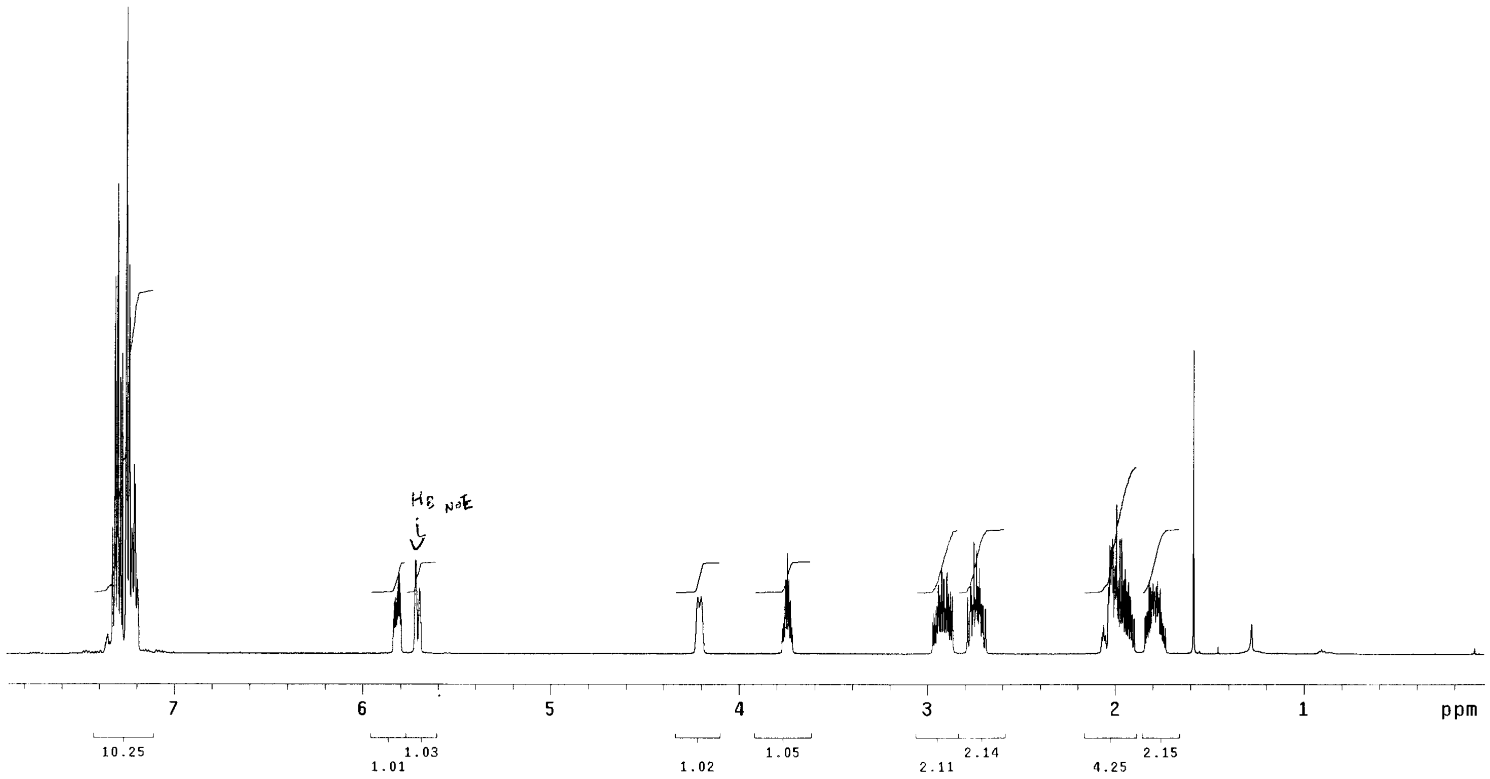

ef003-184

Pulse Sequence: s2pul
Solvent: CDCl3
Ambient temperature
INOVA-500 "Sn.Chem.LSA.UMich.Edu"
PULSE SEQUENCE
Relax. delay 1.000 sec
Pulse 44.2 degrees
Acq. time 2.500 sec
Width 8000.0 Hz
8 repetitions
OBSERVE H1, 499.9042708 MHz
DATA PROCESSING
FT size 65536
Total time 0 min, 28 sec

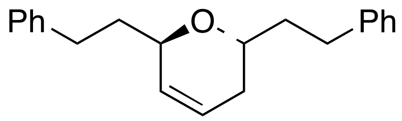

ent-**3a**

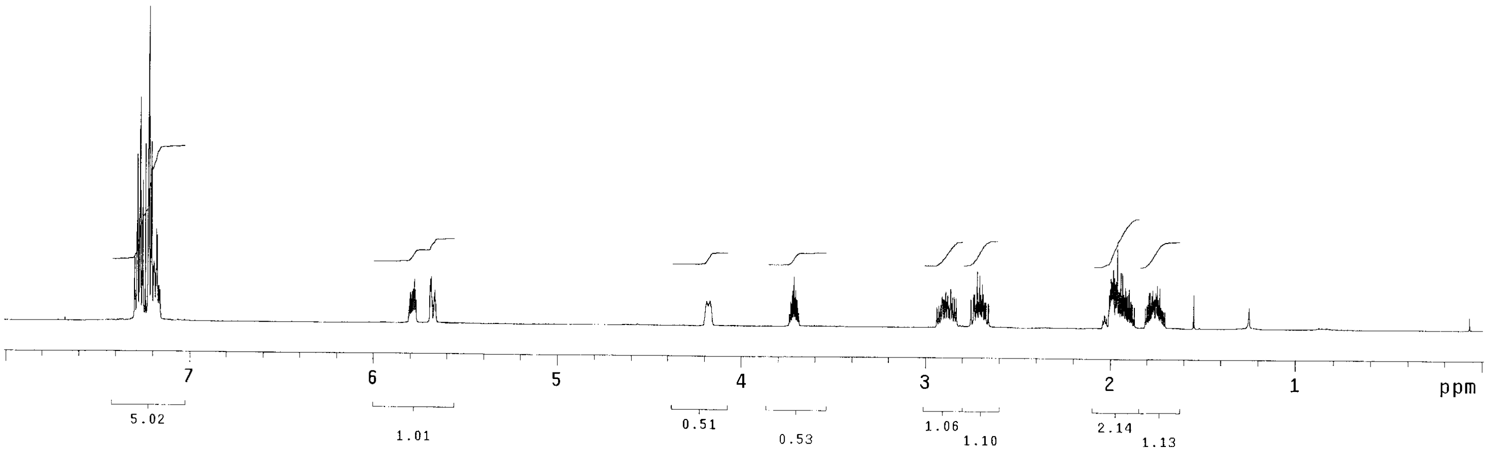

ef003-201

Pulse Sequence: s2pul

Solvent: CDCl3
Ambient temperature
INOVA-500 "Sn.Chem.LSA.UMich.Edu"

PULSE SEQUENCE
Relax. delay 1.000 sec
Pulse 44.2 degrees
Acq. time 2.500 sec
Width 8000.0 Hz
8 repetitions
OBSERVE H1, 499.9042705 MHz
DATA PROCESSING
FT size 65536
Total time 0 min, 28 sec

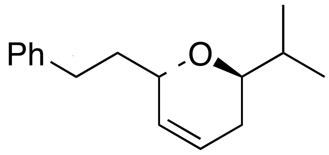

3b

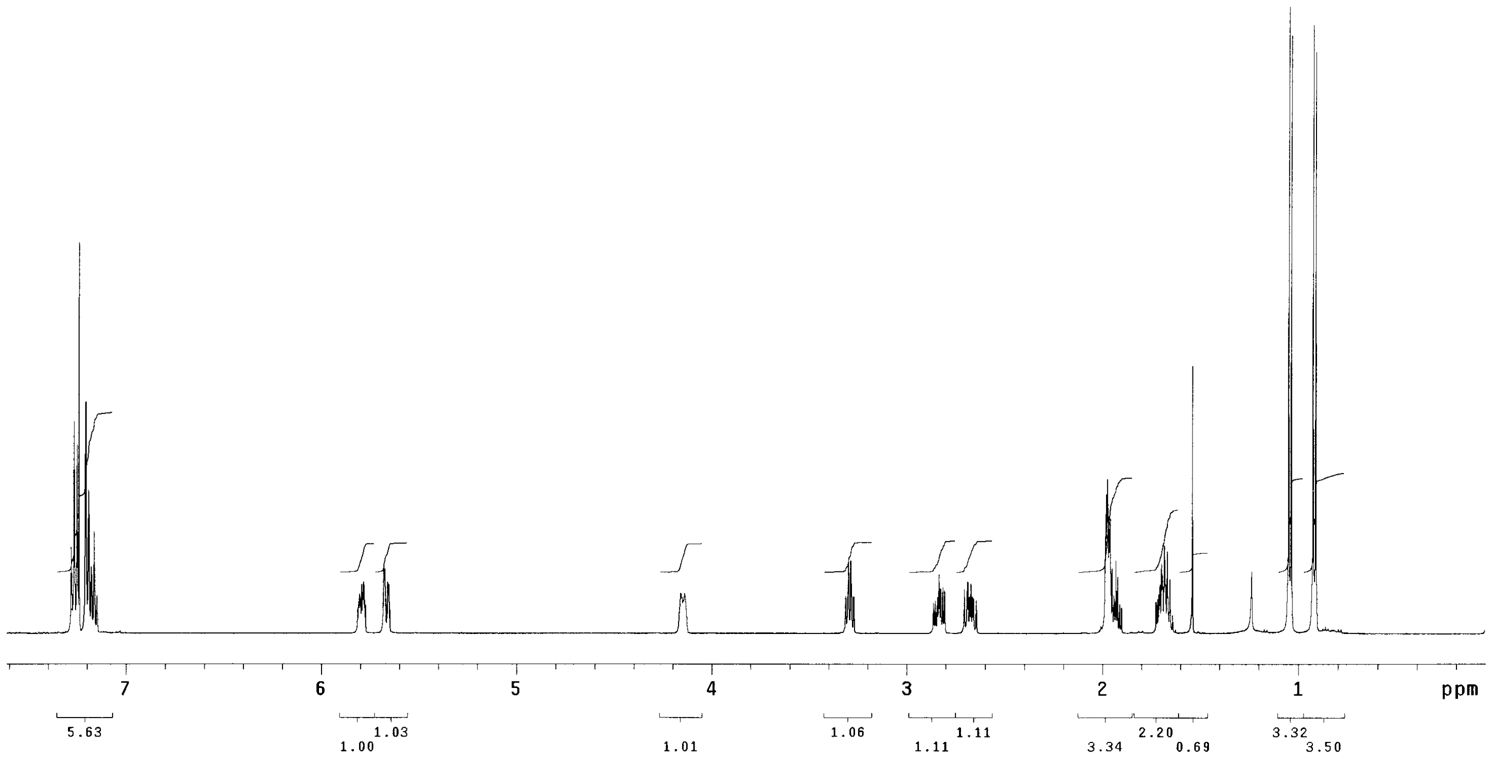

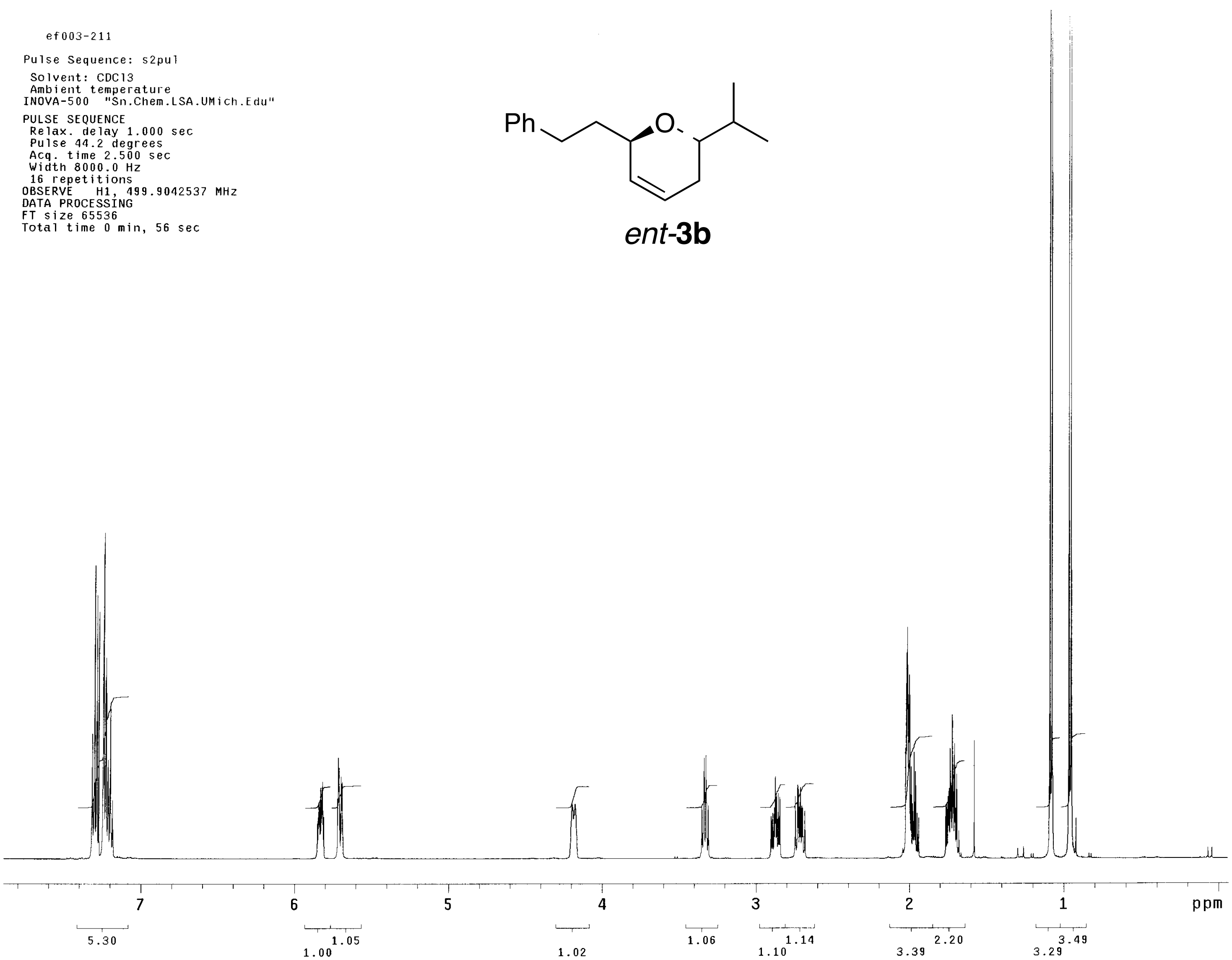
ef003-211
Pulse Sequence: s2pul
Solvent: CDCl3
Ambient temperature
INOVA-500 "Sn.Chem.LSA.UMich.Edu"
PULSE SEQUENCE
Relax. delay 1.000 sec
Pulse 44.2 degrees
Acq. time 2.500 sec
Width 8000.0 Hz
16 repetitions
OBSERVE H1, 499.9042537 MHz
DATA PROCESSING
FT size 65536
Total time 0 min, 56 sec
Ph
O
7
6
5
4
3
2
1
ppm
5.30
1.00
1.05
1.02
1.06
1.10
1.14
3.39
2.20
3.29
3.49

ent-**3b**

ef005-93procut

Pulse Sequence: s2pul
Solvent: CDCl3
Ambient temperature
File: ef005-093
INOVA-500 "Sn"

PULSE SEQUENCE
Relax. delay 1.000 sec
Pulse 44.2 degrees
Acq. time 2.500 sec
Width 8000.0 Hz
8 repetitions
OBSERVE H1, 499.9042705 MHz
DATA PROCESSING
FT size 65536
Total time 0 min, 28 sec

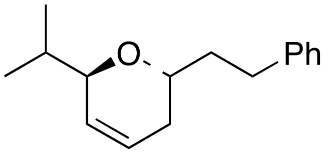

ent-**3c**

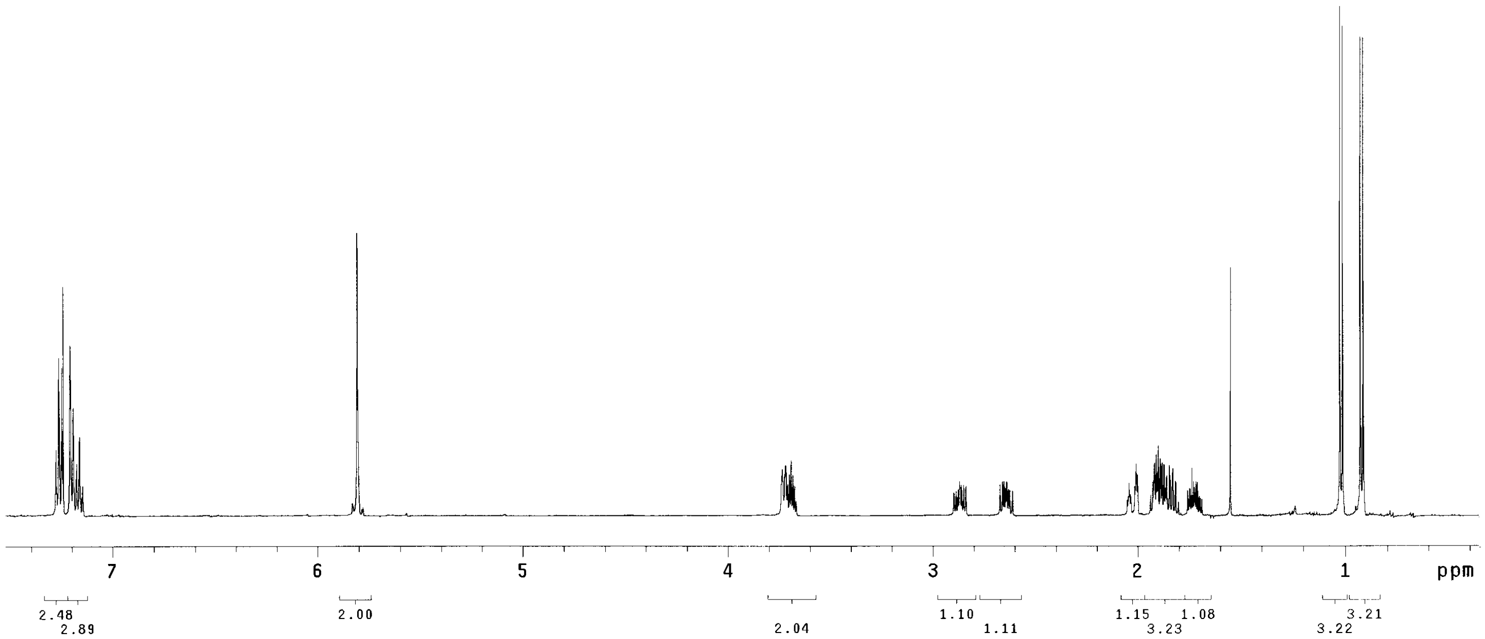

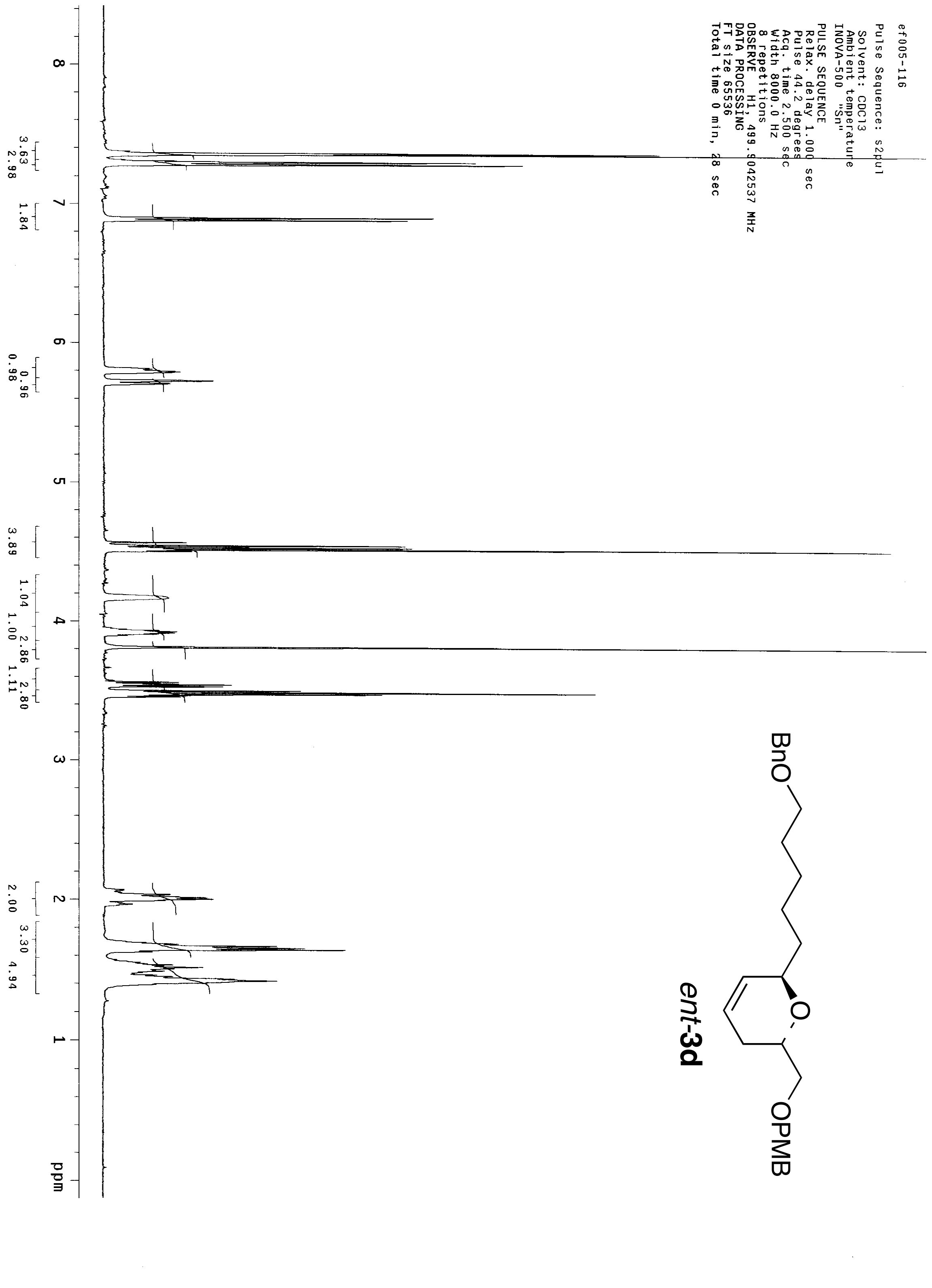
ef005-116
Pulse Sequence: s2pul
Solvent: CDCl3
Ambient temperature
INOVA-500 "Sn"
PULSE SEQUENCE
Relax. delay 1.000 sec
Pulse 44.2 degrees
Acq. time 2.500 sec
Width 8000.0 Hz
8 repetitions
OBSERVE H1, 499.9042537 MHz
DATA PROCESSING
FT size 65536
Total time 0 min, 28 sec
BnO
O
OPMB
ent-3d
3.63
2.98
1.84
0.96
0.98
3.89
1.04
1.00
2.86
1.11
2.80
2.00
3.30
4.94
8
7
6
5
4
3
2
1
ppm

STANDARD PROTON PARAMETERS

Pulse Sequence: s2pul

Solvent: CDCl3
Ambient temperature
INOVA-500 "Sn"

PULSE SEQUENCE
Relax. delay 1.000 sec
Pulse 44.2 degrees
Acq. time 2.500 sec
Width 8000.0 Hz
8 repetitions
OBSERVE H1, 499.9042705 MHz
DATA PROCESSING
FT size 65536
Total time 0 min, 28 sec

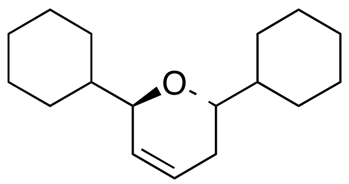

ent-**3e**

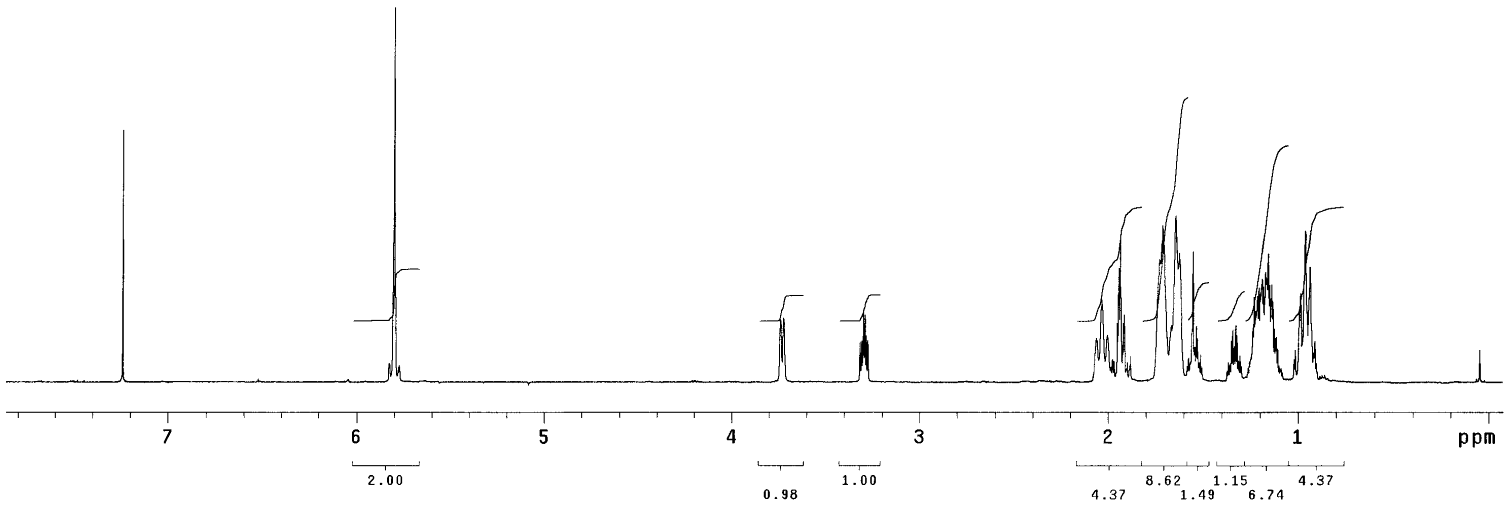

```
ef005-170

Pulse Sequence: s2pul
 Solvent: CDCl3
 Ambient temperature
INOVA-500  "Sn"
PULSE SEQUENCE
 Relax. delay 1.000 sec
 Pulse 42.7 degrees
 Acq. time 2.500 sec
 Width 8000.0 Hz
 8 repetitions
OBSERVE   H1, 499.9042537 MHz
DATA PROCESSING
FT size 65536
Total time 0 min, 28 sec
```

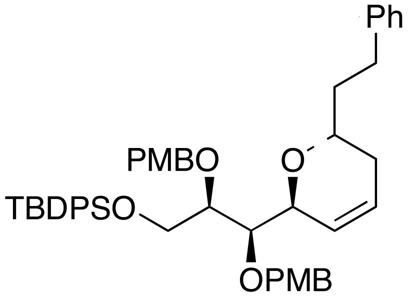

ent-**3f**

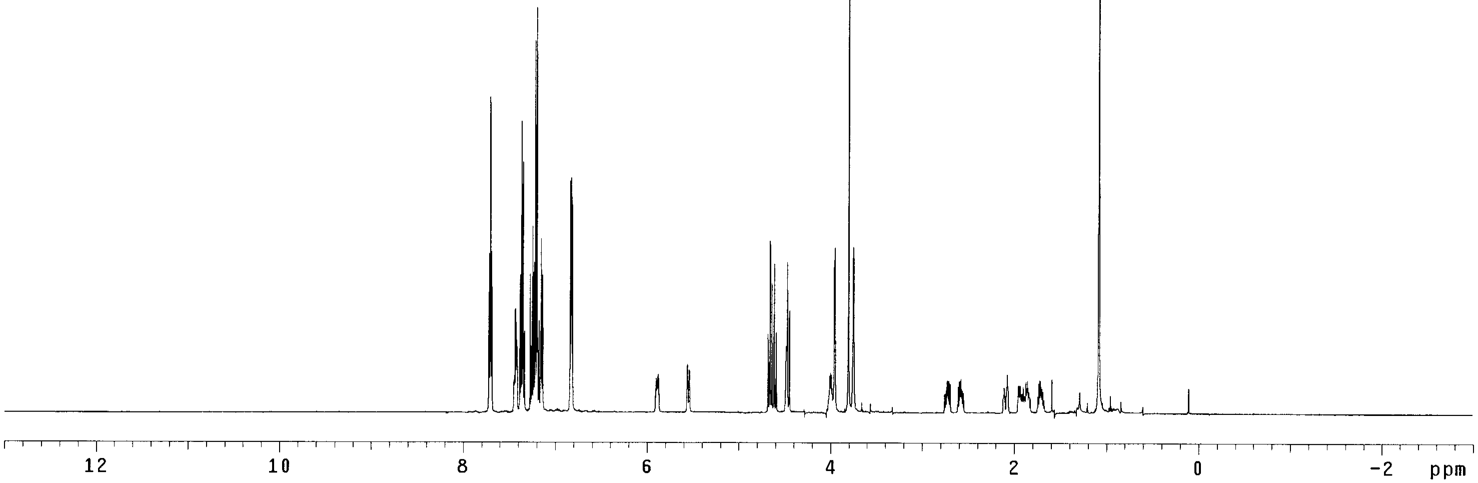

ef003-158 top product isomoer

Pulse Sequence: s2pul

Solvent: CDCl3
Ambient temperature
INOVA-500 "Sn.Chem.LSA.UMich.Edu"

PULSE SEQUENCE
Relax. delay 1.000 sec
Pulse 42.3 degrees
Acq. time 2.500 sec
Width 8000.0 Hz
16 repetitions
OBSERVE H1, 499.9042705 MHz
DATA PROCESSING
FT size 65536
Total time 0 min, 56 sec

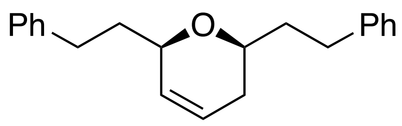

6a

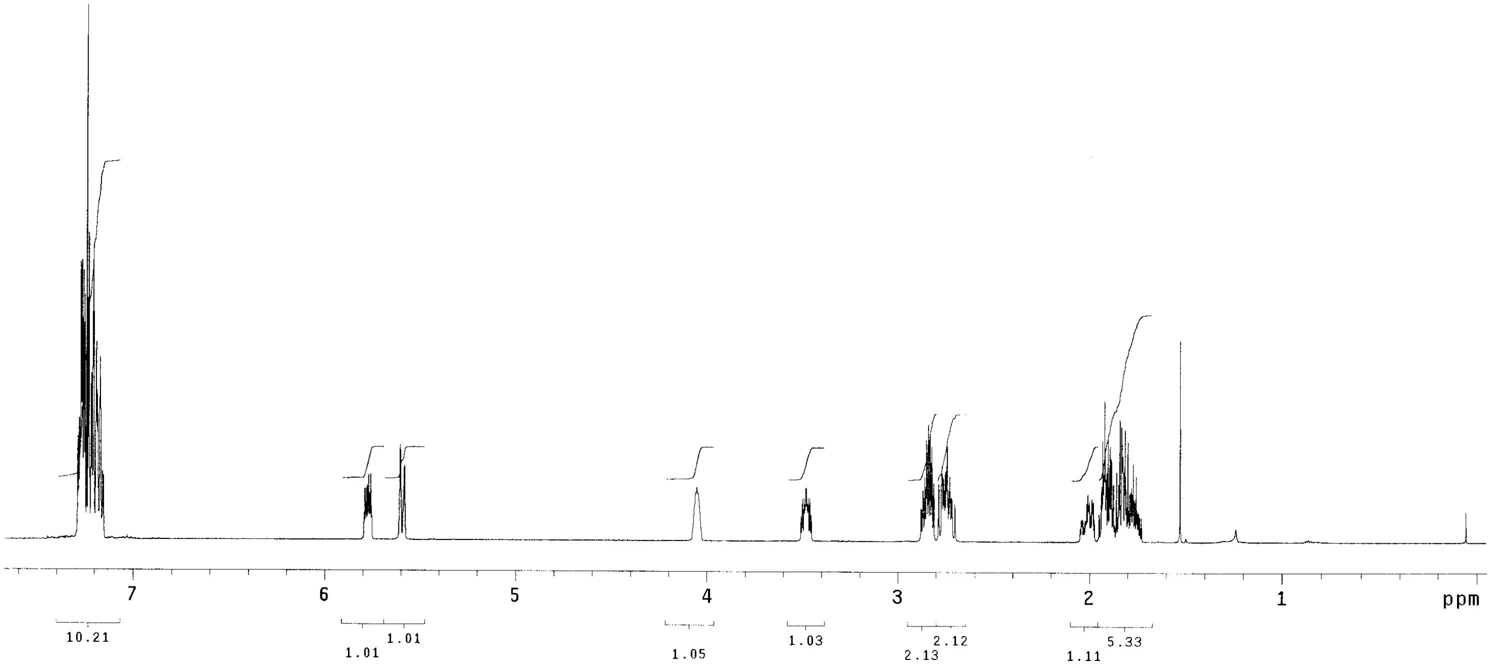

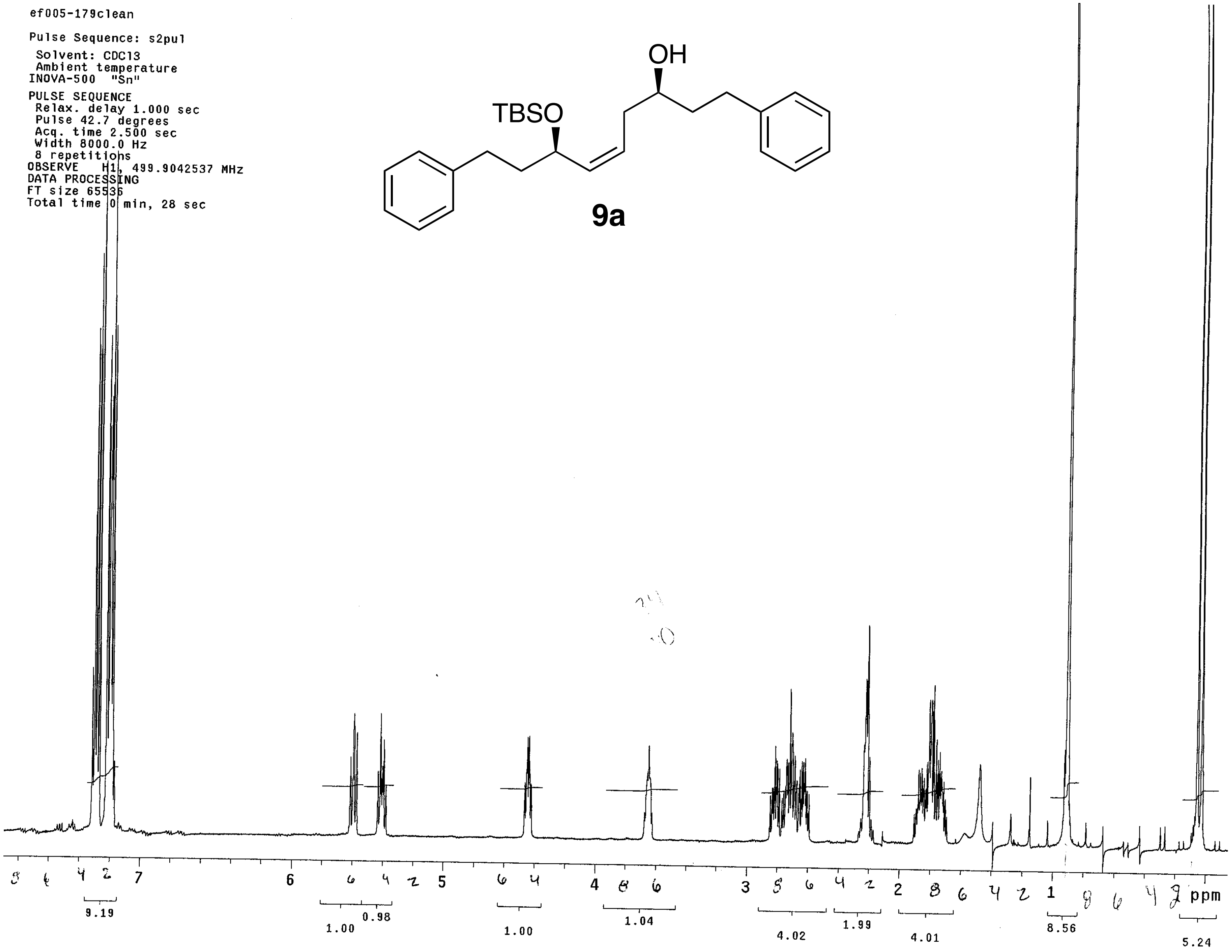
ef005-179clean
Pulse Sequence: s2pul
Solvent: CDCl3
Ambient temperature
INOVA-500 "Sn"
PULSE SEQUENCE
Relax. delay 1.000 sec
Pulse 42.7 degrees
Acq. time 2.500 sec
Width 8000.0 Hz
8 repetitions
OBSERVE H1, 499.9042537 MHz
DATA PROCESSING
FT size 65536
Total time 0 min, 28 sec
TBSO
OH
9a
7
6
5
4
3
2
1
ppm
9.19
1.00
0.98
1.00
1.04
4.02
1.99
4.01
8.56
5.24

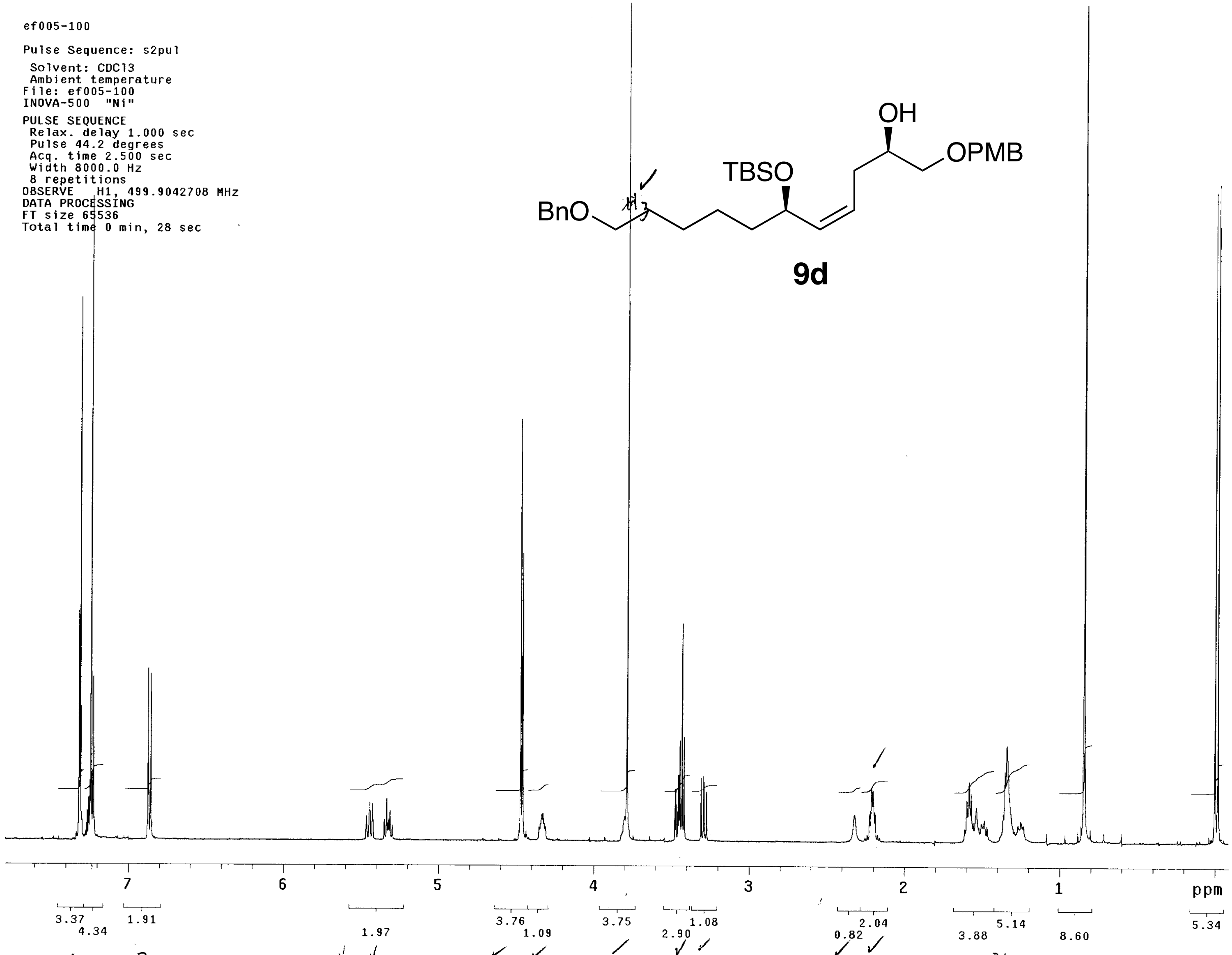
ef005-100
Pulse Sequence: s2pul
Solvent: CDCl3
Ambient temperature
File: ef005-100
INOVA-500 "Ni"
PULSE SEQUENCE
Relax. delay 1.000 sec
Pulse 44.2 degrees
Acq. time 2.500 sec
Width 8000.0 Hz
8 repetitions
OBSERVE H1, 499.9042708 MHz
DATA PROCESSING
FT size 65536
Total time 0 min, 28 sec
BnO
TBSO
OH
OPMB
9d
ppm
3.37
4.34
1.91
1.97
3.76
1.09
3.75
2.90
1.08
0.82
2.04
3.88
5.14
8.60
5.34

ef005091 hplc spot 1

Pulse Sequence: s2pul
Solvent: CDCl3
Ambient temperature
INOVA-500 "Sn"
PULSE SEQUENCE
Relax. delay 1.000 sec
Pulse 44.2 degrees
Acq. time 2.500 sec
Width 8000.0 Hz
8 repetitions
OBSERVE H1, 499.9042537 MHz
DATA PROCESSING
FT size 65536
Total time 0 min, 28 sec

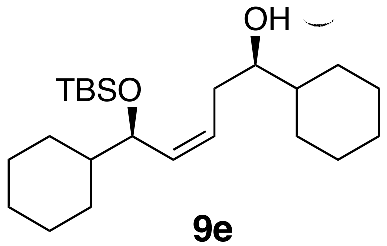

9e

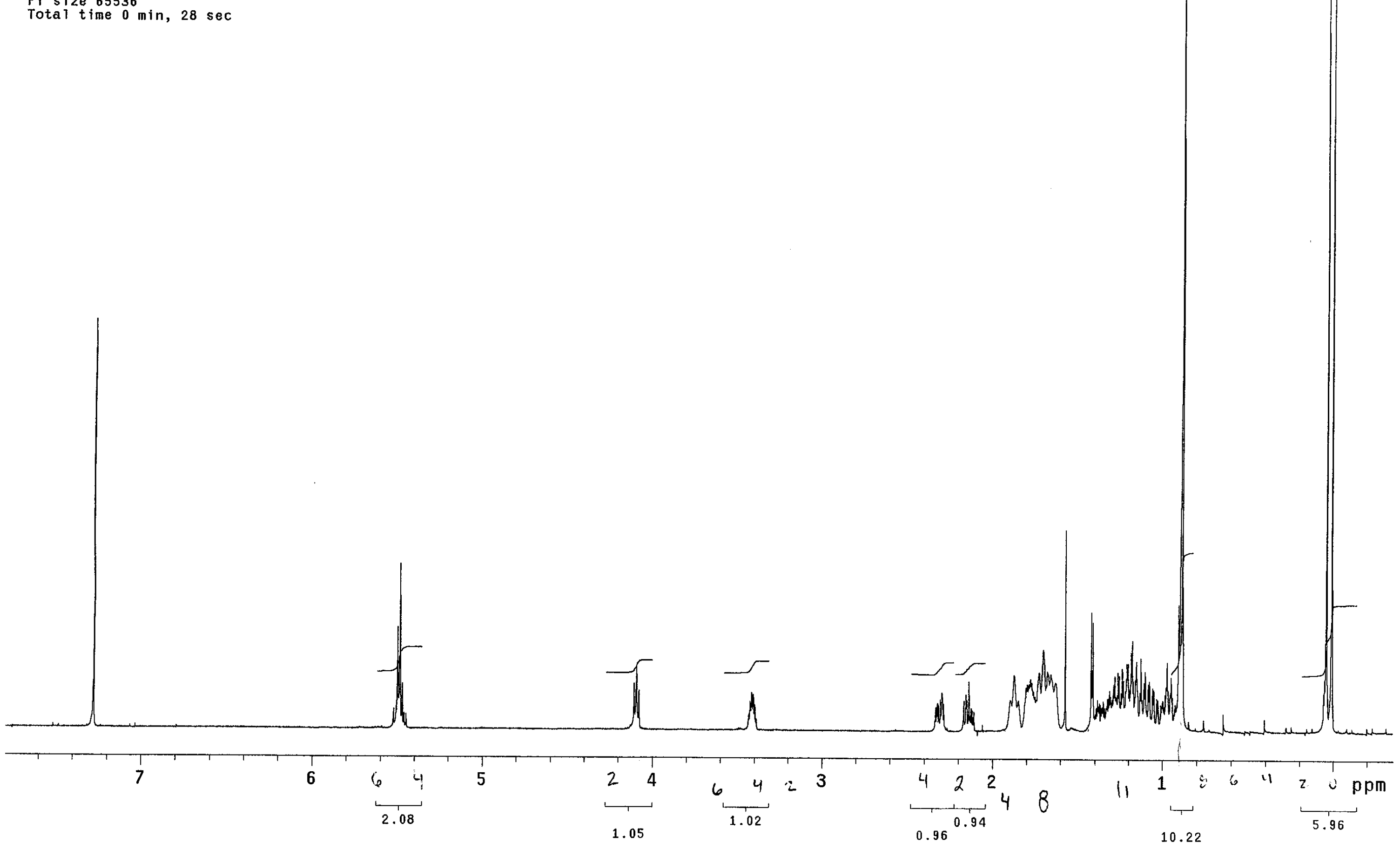

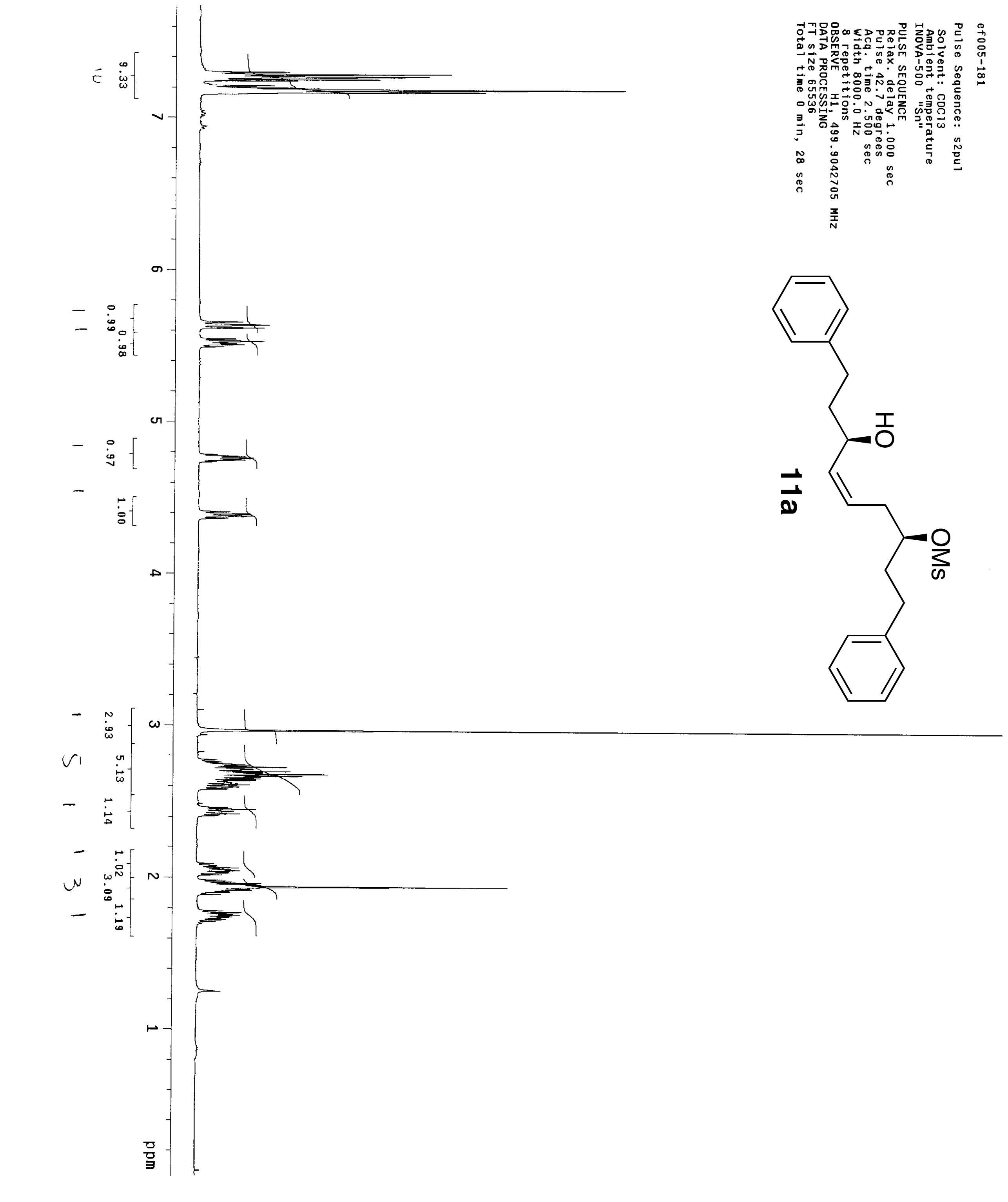
ef005-181
Pulse Sequence: s2pul
Solvent: CDCl3
Ambient temperature
INOVA-500 "Sn"
PULSE SEQUENCE
Relax. delay 1.000 sec
Pulse 42.7 degrees
Acq. time 2.500 sec
Width 8000.0 Hz
8 repetitions
OBSERVE H1, 499.9042705 MHz
DATA PROCESSING
FT size 65536
Total time 0 min, 28 sec
HO
OMs
9.33
0.99
0.98
0.97
1.00
2.93
5.13
1.14
1.02
3.09
1.19
7
6
5
4
3
2
1
ppm

11a

```
ef003-222

Pulse Sequence: s2pul
 Solvent: CDCl3
 Ambient temperature
INOVA-500  "Sn.Chem.LSA.UMich.Edu"
PULSE SEQUENCE
 Relax. delay 1.000 sec
 Pulse 44.2 degrees
 Acq. time 2.500 sec
 Width 8000.0 Hz
 16 repetitions
OBSERVE   H1, 499.9042537 MHz
DATA PROCESSING
FT size 65536
Total time 0 min, 56 sec
```

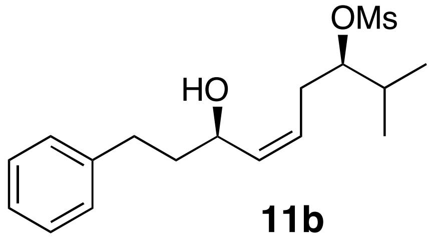

11b

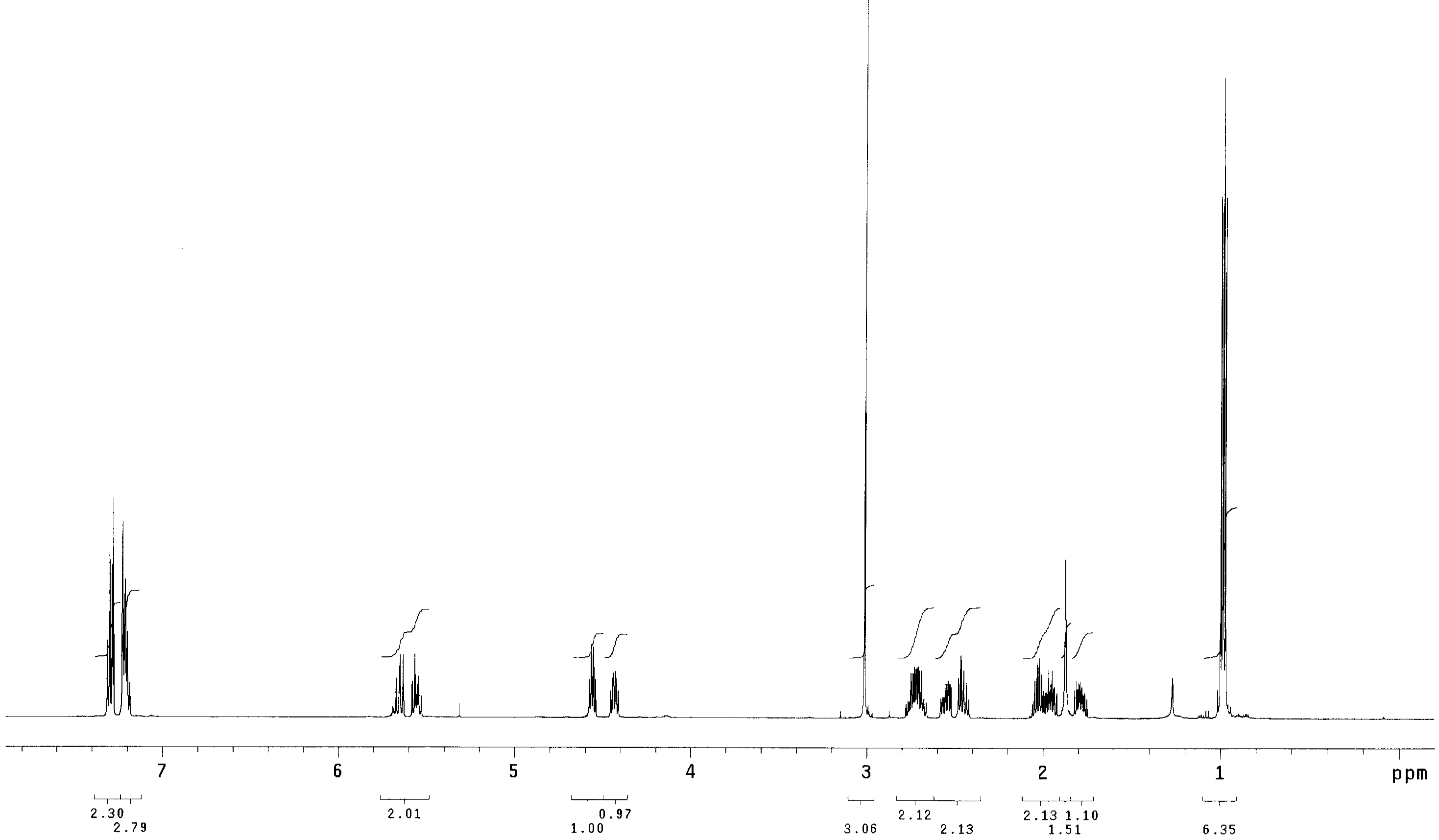

ef005-90

Pulse Sequence: s2pul
Solvent: CDCl3
Ambient temperature
INOVA-500 "Sn"
PULSE SEQUENCE
Relax. delay 1.000 sec
Pulse 44.2 degrees
Acq. time 2.500 sec
Width 8000.0 Hz
8 repetitions
OBSERVE H1, 499.9042705 MHz
DATA PROCESSING
FT size 65536
Total time 0 min, 28 sec

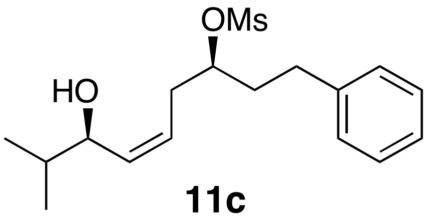

11c

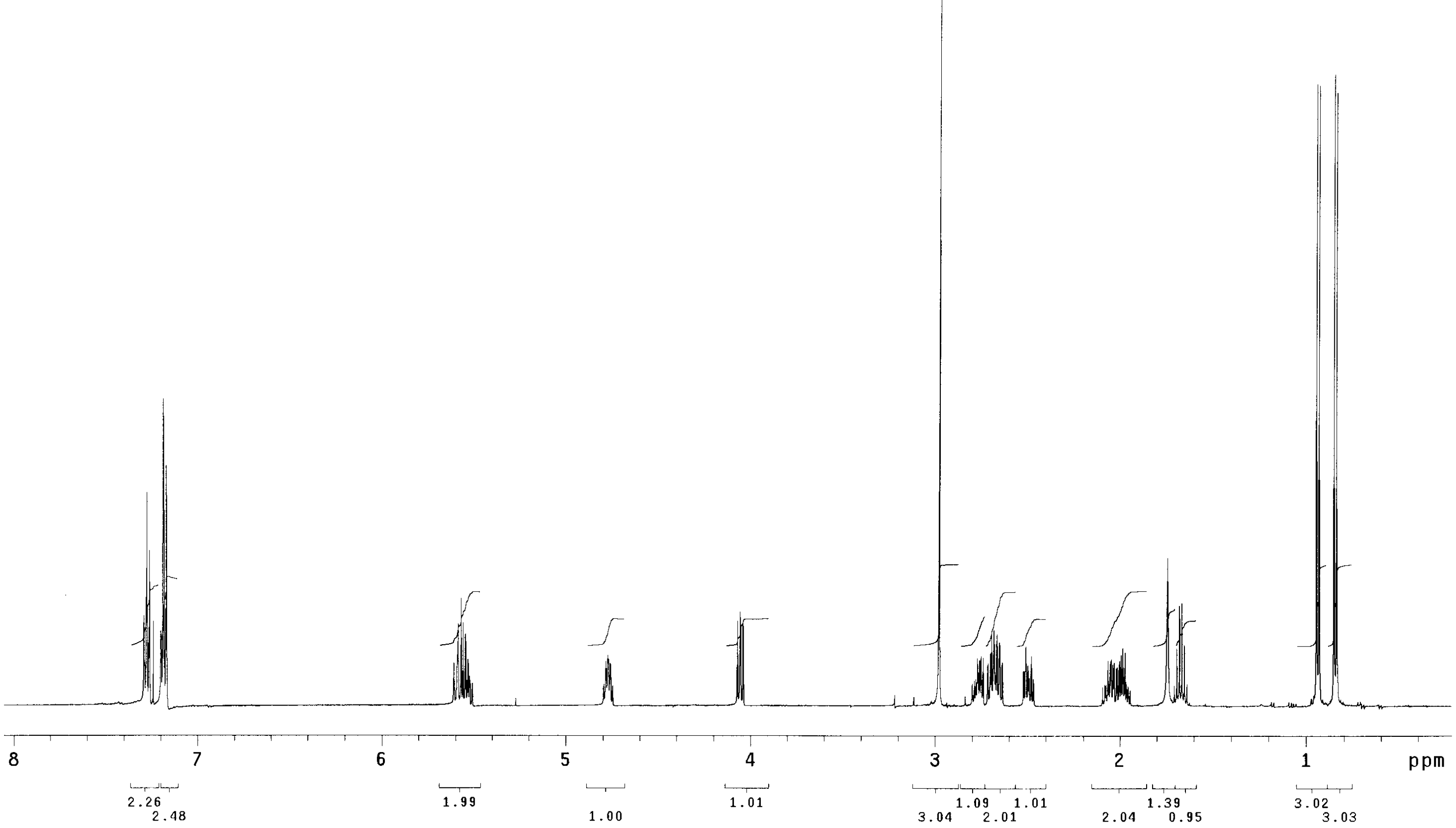

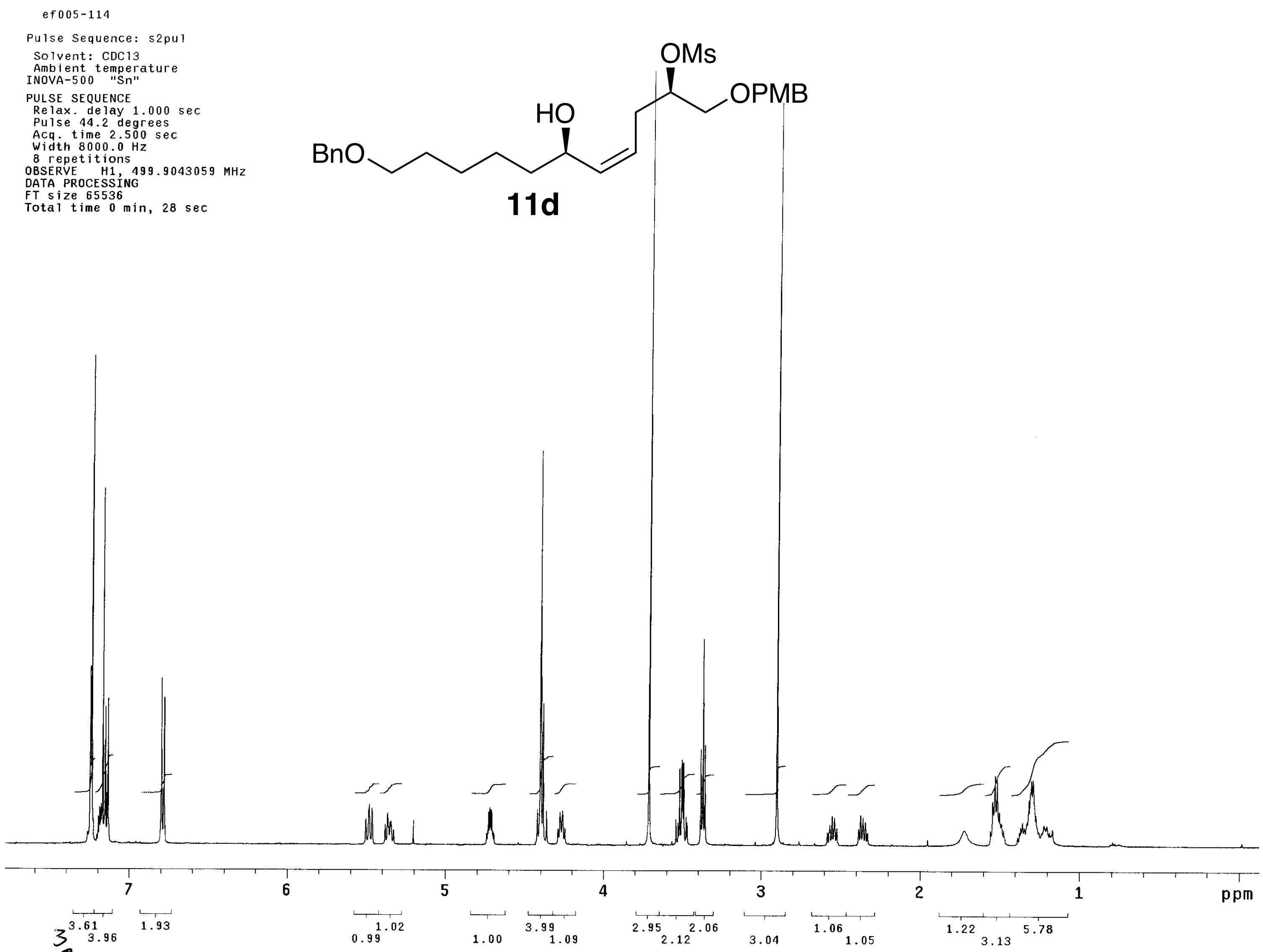
ef005-114
Pulse Sequence: s2pul
Solvent: CDCl3
Ambient temperature
INOVA-500 "Sn"
PULSE SEQUENCE
Relax. delay 1.000 sec
Pulse 44.2 degrees
Acq. time 2.500 sec
Width 8000.0 Hz
8 repetitions
OBSERVE H1, 499.9043059 MHz
DATA PROCESSING
FT size 65536
Total time 0 min, 28 sec
BnO
HO
OMs
OPMB
11d
7
6
5
4
3
2
1
ppm
3.61
3.96
1.93
1.02
0.99
1.00
3.99
1.09
2.95
2.06
2.12
3.04
1.06
1.05
1.22
3.13
5.78

ef005-107

Pulse Sequence: s2pul
Solvent: CDCl3
Ambient temperature
INOVA-500 "Sn"
PULSE SEQUENCE
Relax. delay 1.000 sec
Pulse 44.2 degrees
Acq. time 2.500 sec
Width 8000.0 Hz
8 repetitions
OBSERVE H1, 499.9042708 MHz
DATA PROCESSING
FT size 65536
Total time 0 min, 28 sec

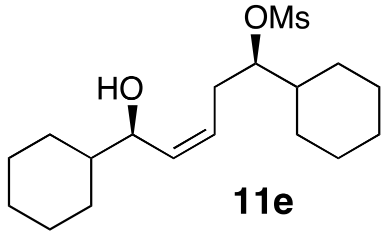

11e

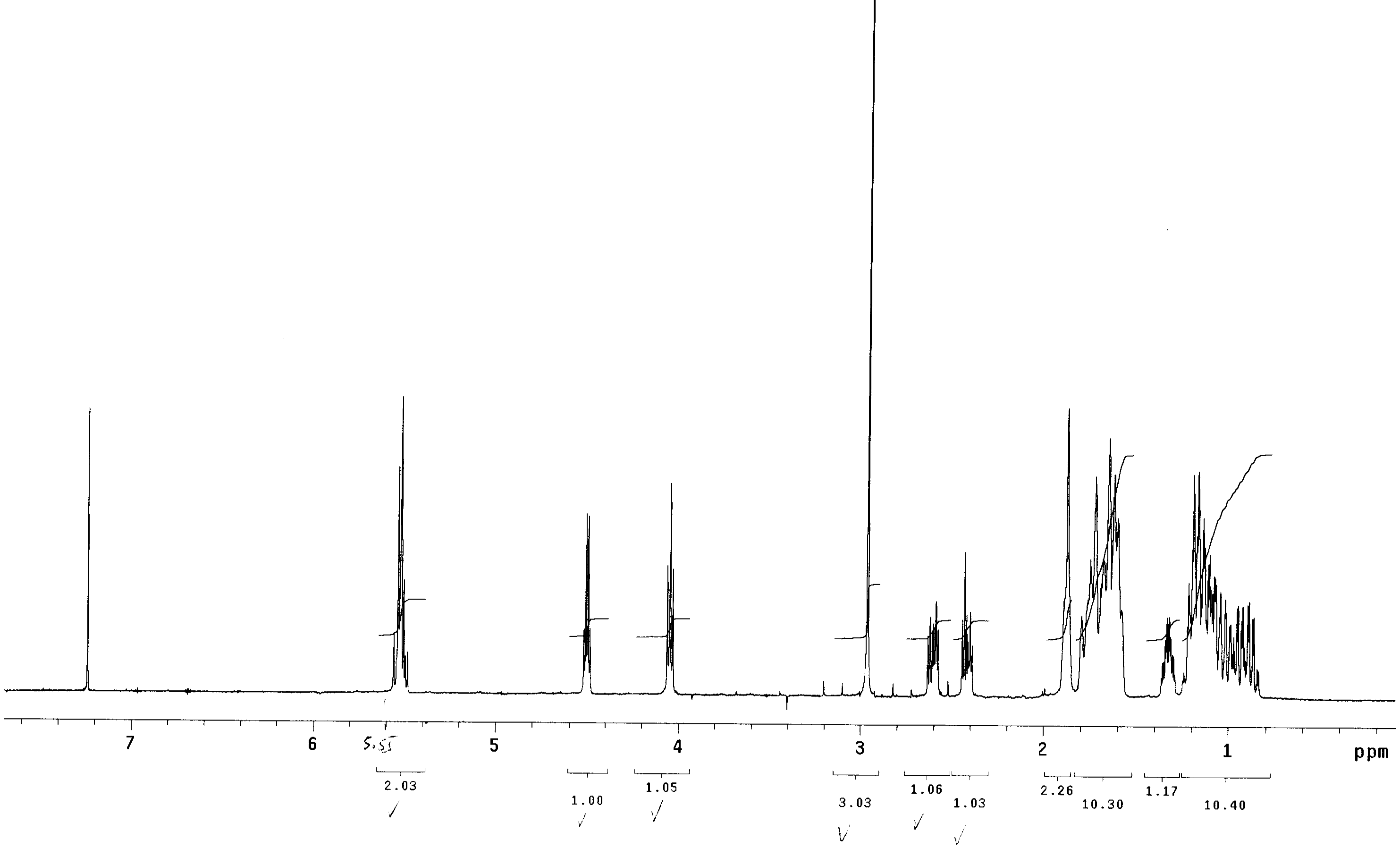

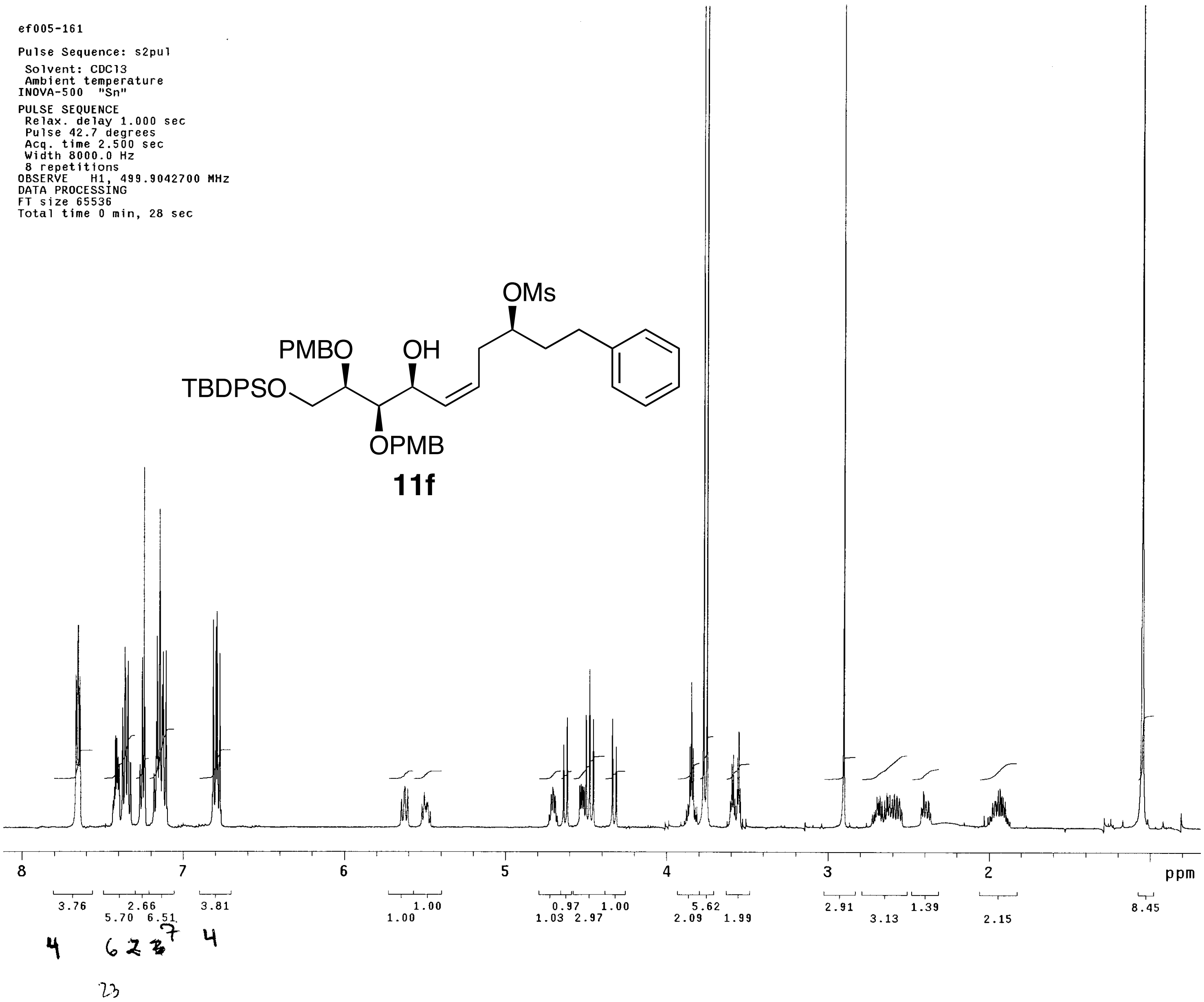
ef005-161
Pulse Sequence: s2pul
Solvent: CDCl3
Ambient temperature
INOVA-500 "Sn"
PULSE SEQUENCE
Relax. delay 1.000 sec
Pulse 42.7 degrees
Acq. time 2.500 sec
Width 8000.0 Hz
8 repetitions
OBSERVE H1, 499.9042700 MHz
DATA PROCESSING
FT size 65536
Total time 0 min, 28 sec
TBDPSO
PMBO
OH
OPMB
OMs
11f
8
7
6
5
4
3
2
ppm
3.76
2.66
5.70
6.51
3.81
1.00
1.00
0.97
1.03
2.97
1.00
5.62
2.09
1.99
2.91
3.13
1.39
2.15
8.45
4
4
